# Pancreatic Cancer and Immunotherapy: A Clinical Overview

**DOI:** 10.3390/cancers13164138

**Published:** 2021-08-17

**Authors:** Florentine E. F. Timmer, Bart Geboers, Sanne Nieuwenhuizen, Madelon Dijkstra, Evelien A. C. Schouten, Robbert S. Puijk, Jan J. J. de Vries, M. Petrousjka van den Tol, Anna M. E. Bruynzeel, Mirte M. Streppel, Johanna W. Wilmink, Hans J. van der Vliet, Martijn R. Meijerink, Hester J. Scheffer, Tanja D. de Gruijl

**Affiliations:** 1 Department of Radiology and Nuclear Medicine, Amsterdam University Medical Centers, De Boelelaan 1117, 1081HV Amsterdam, The Netherlands; b.geboers@amsterdamumc.nl (B.G.); s.nieuwenhuizen1@amsterdamumc.nl (S.N.); m.dijkstra3@amsterdamumc.nl (M.D.); e.schouten@amsterdamumc.nl (E.A.C.S.); r.puijk@amsterdamumc.nl (R.S.P.); j.devries1@amsterdamumc.nl (J.J.J.d.V.); mr.meijerink@amsterdamumc.nl (M.R.M.); hj.scheffer@amsterdamumc.nl (H.J.S.); 2 Department of Surgery, Amsterdam University Medical Centers, De Boelelaan 1117, 1081HV Amsterdam, The Netherlands; mp.vandentol@amsterdamumc.nl; 3 Department of Radiation Oncology, Amsterdam University Medical Centers, De Boelelaan 1117, 1081HV Amsterdam, The Netherlands; ame.bruynzeel@amsterdamumc.nl; 4 Department of Medical Oncology, Cancer Center Amsterdam, Amsterdam University Medical Centers, De Boelelaan 1117, 1081HV Amsterdam, The Netherlands; m.m.streppel@amsterdamumc.nl (M.M.S.); j.w.wilmink@amsterdamumc.nl (J.W.W.); jj.vandervliet@amsterdamumc.nl (H.J.v.d.V.); td.degruijl@amsterdamumc.nl (T.D.d.G.); 5 LAVA Therapeutics, Yalelaan 60, 3584CM Utrecht, The Netherlands

**Keywords:** pancreatic cancer, immunotherapy, immunomodulators, oncolytic virus, adoptive cell therapy, cancer vaccine, ablation

## Abstract

**Simple Summary:**

Pancreatic ductal adenocarcinoma (PDAC) is an aggressive cancer with a dismal prognosis. While immunotherapy has been deemed a breakthrough treatment for various subtypes of cancer, its efficacy in PDAC is limited. This review discusses a wide range of immunotherapies, providing a general introduction to their working mechanism as well as current evidence on their clinical efficacy and immune eliciting abilities in PDAC. Utilizing combination (immuno)therapies to generate synergistic anti-tumor effects may provide the key to successful PDAC treatment.

**Abstract:**

Pancreatic ductal adenocarcinoma (PDAC) is an aggressive disease with high mortality. The vast majority of patients present with unresectable, advanced stage disease, for whom standard of care chemo(radio)therapy may improve survival by several months. Immunotherapy has led to a fundamental shift in the treatment of several advanced cancers. However, its efficacy in PDAC in terms of clinical benefit is limited, possibly owing to the immunosuppressive, inaccessible tumor microenvironment. Still, various immunotherapies have demonstrated the capacity to initiate local and systemic immune responses, suggesting an immune potentiating effect. In this review, we address PDAC’s immunosuppressive tumor microenvironment and immune evasion methods and discuss a wide range of immunotherapies, including immunomodulators (i.e., immune checkpoint inhibitors, immune stimulatory agonists, cytokines and adjuvants), oncolytic viruses, adoptive cell therapies (i.e., T cells and natural killer cells) and cancer vaccines. We provide a general introduction to their working mechanism as well as evidence of their clinical efficacy and immune potentiating abilities in PDAC. The key to successful implementation of immunotherapy in this disease may rely on exploitation of synergistic effects between treatment combinations. Accordingly, future treatment approaches should aim to incorporate diverse and novel immunotherapeutic strategies coupled with cytotoxic drugs and/or local ablative treatment, targeting a wide array of tumor-induced immune escape mechanisms.

## 1. Introduction

Pancreatic ductal adenocarcinoma (PDAC) is an aggressive disease, with a dismal five-year overall survival (OS) rate of 6% [1]. Symptoms are often nonspecific with a late clinical onset, allowing the tumor to progress freely and silently. As a result, over 80% of patients present with unresectable locally advanced (LAPC) or metastatic PDAC (mPDAC) [2,3]. In these patients, survival can be moderately improved with palliative-intent systemic chemotherapy consisting of gemcitabine/nab-paclitaxel or FOLFIRINOX (folinic acid, 5-fluorouracil, irinotecan and oxaliplatin) [4,5].

**Figure 1:**
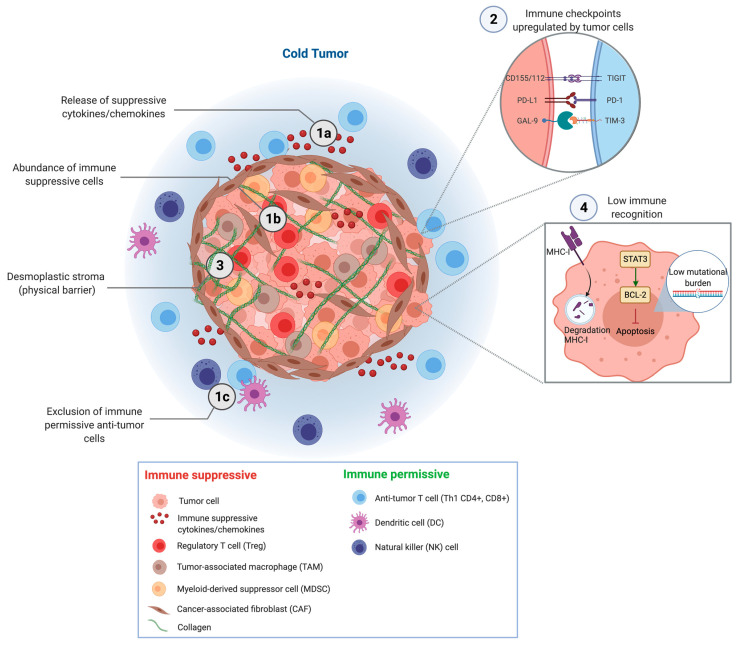
The immune evasion mechanisms of pancreatic cancer. (1a) Tumor cells release a plethora of immune-suppressive, pro-tumorigenic cytokines (e.g., IL-6, IL-8, IL-10, TGF-β, M-CSF and VEGF) and chemokines (e.g., CXCL12) into the microenvironment, which (1b) attract and activate immune-suppressive cells (including Tregs, MDSCs, TAMs and CAFs), subsequently resulting in (1c) exclusion of immune permissive anti-tumor cells (including Th1 CD4^+^ cells, CD8^+^ T cells, DCs and NK cells). (2) Tumor cells upregulate co-inhibitory receptors, or immune checkpoints, such as PD-L1, CD155/112 and Gal-9, to impede an anti-tumor T cell response. (3) Tumor-induced CAFs modulate the extracellular matrix, promoting fibrotic reformation, leading to a desmoplastic stroma which acts as a physical barrier for anti-tumor immune cells and systemic treatments. (4) Tumor cells increase their apoptotic resistance through augmented expression of apoptotic regulatory proteins STAT3 and BCL-2. Reduced immune recognition is established by its low mutational burden and through downregulation of MHC-I membrane proteins.

Immunotherapy has led to a paradigm shift in the treatment of a subset of various solid cancers. However, in PDAC the currently available immunotherapies have only demonstrated marginal efficacy in terms of survival [6,7]. The treatment insensitivity of PDAC to immunotherapies can be attributed to the low mutational burden as well as the densely packed, inaccessible tumor microenvironment (TME) with fibrotic, hypoxic and immunosuppressive features, rendering the tumor immunologically ‘cold’ (Figure 1) [8]. Nonetheless, systemic and local immune responses have been reported in PDAC patients after treatment with immunotherapy, implying they can instigate an anti-tumor effect [9], albeit not potent enough. In this regard, combination with other immunotherapies, chemotherapy and/or local ablation may work synergistically. Certain cytotoxic drugs and ablative therapies have demonstrated the ability to sensitize the TME to immunotherapeutic agents by inducing immunogenic cell death, disrupting immune evasive mechanisms and reducing immune suppression [10,11]. Here, we provide an overview of currently available immunotherapies and their anti-tumor effects in PDAC in terms of immune activation and survival, with a focus on clinical research from the past decade. Additionally, we explore the potential of multimodal treatment strategies and illustrate how synergistic combinations may provide the key to successful PDAC therapy.

## 2. Mechanisms of Immune Evasion

The pancreatic TME is considered extremely immunosuppressive and generally lacking in immune infiltration (Figure 1) [12]. Poor infiltration by T cells may in part be attributed to the low mutational burden of PDAC and consequent lack of neoantigens, which serve as immune targeting molecules and have the ability to elicit high-affinity T cell responses [13]. Further immune suppression and limited anti-tumor T cell infiltration is achieved through various mechanisms that enable immune evasion and immune exclusion from the TME. The TME comprises extracellular matrix (ECM), cancerous cells and non-cancerous cells including (mainly) pro-tumorigenic stromal, immune and endothelial cells. The cancerous cells release pro-tumorigenic chemokines, such as C-X-C motif chemokine ligand (CXCL)12, and pro-tumorigenic cytokines, including interleukin (IL)-6, IL-8, IL-10, transforming growth factor-beta (TGF-β), macrophage colony-stimulating factor (M-CSF) and vascular endothelial growth factor (VEGF) [14,15]. Abundant release of these molecules sways the immune balance from effective immune surveillance to immune tolerance by activating regulatory T cells (Tregs), tumor-associated macrophages (TAMs), T-helper2 (Th2) cells and myeloid-derived suppressor cells (MDSCs). This change in immune status contributes to tumor cell proliferation, migration and angiogenesis [16,17,18]. These immune cell subsets block anti-tumor activity of natural killer (NK) cells and effector CD4^+^ and CD8^+^ T cells. Additionally, maturation and survival of dendritic cells (DCs) is also impeded by these pro-tumorigenic cytokines [19,20]. DCs are powerful antigen presenting cells (APCs) essential in establishing a potent anti-tumor T cell response. Higher DC levels in blood and tumor tissue of PDAC patients have been correlated with improved survival [21,22,23]. Further immune evasion by the tumor is established through downregulation of the antigen presentation machinery, including major histocompatibility complex (MHC) class I [24], upregulation of inhibitory immune checkpoint ligands and increased apoptotic resistance through augmented expression of apoptotic regulatory proteins, signal transducer and activator of transcription (STAT)3 and B-cell lymphoma (BCL)-2 [19,25]. In addition to immune evasion, PDAC is able to physically exclude anti-tumor immune cells from its TME through activation of cancer-associated fibroblasts (CAFs) [12]. These CAFs promote fibrosis through collagen deposition and rearrangements of the ECM, leading to a desmoplastic reaction. The desmoplastic stroma, comprising up to 50–80% of tumor volume, creates a physical barrier preventing adequate vascularization, hindering infiltration of anti-tumor immune cells and resisting systemic treatments [26,27]. This barrier is present in primary as well as metastatic pancreatic tumors [28]. Collectively, these mechanisms contribute to an overall immune-inhibitory PDAC TME and explain the limited response to both chemo- and immunotherapy [29].

## 3. Regulation of the T Cell Response

To establish an anti-tumor immune response, both a priming phase and effector phase are required. In the priming phase, APCs present the acquired tumor antigens to T cells, initiating clonal expansion of antigen-specific T cells. The effector phase covers the direct anti-tumor T cell activity. T cell activation in both phases requires T cell receptor (TCR) complex ligation with antigen-loaded MHC class I (expressed on all nucleated cells) or class II (expressed on APCs). This is known as signal 1. However, signal 1 by itself is ineffective at T cell activation. Co-stimulation through specialized receptors is necessary to fully establish an anti-tumor T cell response, referred to as signal 2. The most critical co-stimulation is provided by binding of cluster of differentiation (CD)80 or CD86 on APCs with CD28 on T cells. Other co-stimulatory receptors include OX40, CD137 (4-1BB), CD27 and inducible T cell co-stimulator (ICOS) on T cells and CD40 on APCs or tumor cells. Their respective ligands are OX40L, CD137L, CD70 and ICOSL on APCs or tumor cells and CD40L on T cells (Table 1). Co-inhibitory receptors, or immune checkpoints, on T cells play an important role in the restriction of inflammatory responses by providing negative feedback upon T cell activation, thus overriding these activation signals. Co-inhibitory receptors include programmed death (PD)-1, cytotoxic T-lymphocyte-associated protein (CTLA)-4, T cell immunoglobulin and mucin domain-containing protein (TIM)-3, T cell immunoglobulin and ITIM domain (TIGIT) and lymphocyte-activation gene (LAG)-3 (Table 1) [30]. Their respective ligands include, but are not limited to, PD-L1/2, CD80/86, galectin (Gal)-9, CD155/112 and MHC-I/II. Tumor cells are able to abuse these regulatory mechanisms, upregulating inhibitory checkpoint ligands on their cellular membrane or mobilizing immune-suppressive myeloid cells with high expression levels of these checkpoint ligands and downregulating activating immune receptors on T cells, thereby avoiding recognition by patrolling immune cells [31].

## 4. Immunotherapy

Immunotherapies can be categorized into immunomodulators (i.e., immune checkpoint inhibitors, immune stimulatory agonists, bispecific antibodies (not discussed in this review), cytokines and adjuvants), oncolytic viruses, adoptive cell therapies (i.e., T cells and NK cells) and cancer vaccines, each with a unique mechanism of action. Below, various immunotherapies are addressed within the context of PDAC. Of note, only a selection of clinical trials listed in the tables will be discussed more in-depth in the text.

### 4.1. Immune Checkpoint Inhibitors

#### 4.1.1. Anti-PD-1/Anti-PD-L1

**Figure 2:**
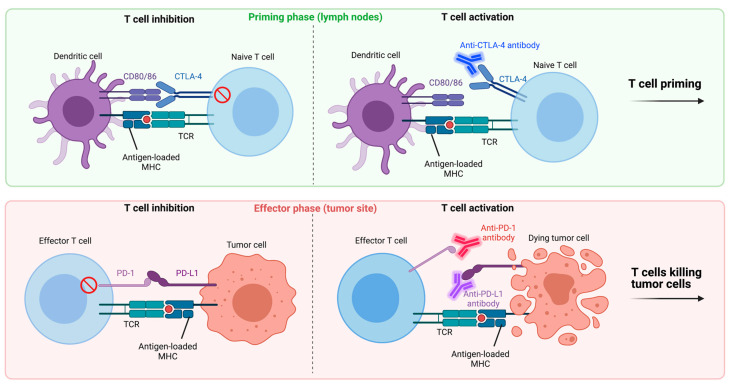
Immune checkpoint inhibitors anti-CTLA-4, anti-PD1 and anti-PD-L1. During the priming phase (in the lymph nodes), T cell priming may be inhibited, even though they are presented with antigen-loaded MHC complexes, through binding of CD80 or CD86 on dendritic cells (DCs) with CTLA-4 on naïve T cells. Anti-CTLA-4 antibodies block this interaction, reversing T cell inhibition, prompting activation and expansion of antigen-specific effector T cells. During the effector phase (at the tumor site), T cell inhibition can be established through PD-1 ligation (T cells) with PD-L1 (tumor and myeloid cells). This process can be reversed by using anti-PD-1 or anti-PD-L1 antibodies, allowing CD8^+^ T cell-induced killing of tumor cells. Of note, recent studies also point to decreases in suppressive Treg rates and decreased T cell priming by CD28 signaling interference upon CTLA-4 and PD-1 blockade, respectively.

PD-1 ligation promotes self-tolerance by inhibiting T cell activation and proliferation (Figure 2) [32]. Moreover, signaling pathways can interfere with T cell activation through signaling interference downstream from the TCR complex and CD28 [33,34] and promote apoptosis [35,36]. The PD-1 pathway can be blocked by immune checkpoint inhibitors anti-PD-1 or anti-PD-L1. If PD-L1 is overexpressed, although uncommon in PDAC [37], it inversely correlates with CD8^+^ TIL frequency and clinical prognosis [38,39,40,41]. Despite encouraging results of anti-PD-1 and anti-PD-L1 therapy in patients with other solid malignancies [42,43], results in patients with PDAC are less impressive. To date, various clinical trials have described the effects of anti-PD-1/anti-PD-L1 in PDAC as monotherapy [44,45,46] or in combination with other systemic therapies [46,47,48,49,50,51,52] (Table 2). The most promising outcomes were reported in a cohort of mPDAC patients (*n* = 17), partially chemotherapy-naïve (*n* = 11), who received anti-PD-1 concurrently with a gemcitabine/nab-paclitaxel regimen, resulting in a median progression-free survival (mPFS) of 9.1 months and median overall survival (mOS) of 15 months [48]. As reference, mOS outcomes of 8.5 and 11.1 months may be achieved with gemcitabine/nab-paclitaxel and FOLFIRINOX, respectively [4,5]. Recently, results from the phase 2a COMBAT trial suggested that chemotherapy concurrent with combined anti-PD-1 and anti-C-X-C motif chemokine receptor (CXCR)4 may augment chemotherapeutic effects after attaining an mOS of 7.8 months from the start of immunotherapy in pre-treated mPDAC patients [49]. Results from both trials are in line with the expanding recognition that cytotoxic drugs (i.e., chemotherapy) can enhance immunotherapeutic effects by stimulating immunogenic tumor cell death, reducing tumor-induced immune suppression and increasing effector T cell function and infiltration [53,54,55]. Definitive advice regarding combined treatment with chemotherapy (gemcitabine/paclitaxel or FOLFIRINOX) and anti-PD-1 may be provided by two large RCTs (ClinicalTrials.gov Identifier: NCT04674956; NCT03983057). In contrast, combining anti-PD-L1 with the Bruton’s tyrosine kinase inhibitor ibrutinib, which targets various oncogenic driver pathways, in pre-treated mPDAC patients (*n* = 48) showed limited anti-tumor activity (mOS 4.2 months, mPFS 1.7 months) [52].

#### 4.1.2. Anti-CTLA-4

On T cells, the inhibitory CTLA-4 receptor competes with co-stimulatory receptor CD28 for the CD80 and CD86 ligands on APCs, for which CTLA-4 has higher affinity [62,63]. Lower CTLA-4 and higher CD80 expression in PDAC are linked to improved survival [39,64]. Ligation of CTLA-4 mainly limits priming of naïve T cells in the lymphoid organs (Figure 2) but may also impede direct anti-tumor T cell activity in the effector phase, possibly through decreasing suppressive Treg rates [62,65,66]. Anti-CTLA-4 therapy has demonstrated moderately favorable results in other malignancies [67,68], but lacks demonstrable beneficial results in PDAC. Several articles have been published on the use of anti-CTLA-4 monotherapy in PDAC [56,57], or combined with other immunotherapeutic agents [46,57] or chemotherapy [58,59,60,61] (Table 2). A series of articles [59,60,61] within a phase 1b clinical trial examined the efficacy of anti-CTLA-4 combined with gemcitabine in advanced PDAC patients (*n* = 21) and reported an mOS of 6.9 months and mPFS of 2.5 months, similar to gemcitabine treatment alone (6.8 months) [4]. Other research noted that adding anti-CTLA-4 to anti-PD-L1 in pre-treated mPDAC patients (*n* = 65) did not improve survival compared to anti-PD-L1 monotherapy (mOS 3.1 vs. 3.6 months; mPFS 1.5 vs. 1.5 months) [46]. Le et al. [57] assessed the value of a granulocyte macrophage colony-stimulating factor (GM-CSF) transduced allogeneic pancreatic cancer cell line-based vaccine (GVAX) added to anti-CTLA-4 in gemcitabine pre-treated advanced PDAC patients. Through randomization, patients were allocated anti-CTLA-4 monotherapy (*n* = 15) or anti-CTLA-4 combined with GVAX (*n* = 15), reporting mOS outcomes of 3.6 and 5.7 months, respectively. Among patients with an OS > 4.3 months, there was an enhancement in mesothelin-specific T cells (*p* = 0.014) and augmentation of the T cell repertoire (*p* = 0.031). Hence, addition of GVAX to anti-CTLA-4 seems to induce a T cell mediated immune response and may slightly improve survival in advanced PDAC patients. Several trials are ongoing that use anti-CTLA-4 treatment combined with other immunotherapies and/or radiotherapy in PDAC (ClinicalTrials.gov Identifier: NCT03104439; NCT04258150; NCT03816358).

#### 4.1.3. Anti-TIM-3

Dysfunctional and (terminally) exhausted CD4^+^ and CD8^+^ T cells as well as tumoral DCs are characterized by overexpression of TIM-3 [69,70], which is likely related to tumor cell invasion, metastatic processes and recurrence [71]. TIM-3 has multiple ligands, including Gal-9, phosphatidylserine (PtdSer), carcinoembryonic antigen-related cell adhesion molecule 1 (CEACAM1) and high mobility group protein B1 (HMGB1), all of which are implicated in its immune inhibitory functions [70,72,73,74]. Ligation of Gal-9, the first known and most studied TIM-3 ligand, with TIM-3 on T cells and NK cells leads to diminished activation and, hence, inhibited anti-tumor activity. Expression of Gal-9 is increased in PDAC tissue, both on tumor and immune cells, as well as in blood of PDAC patients when compared to healthy pancreas tissue and healthy individuals, respectively [75]. There are no (upcoming) data from (pre-)clinical research on the therapeutic effects of anti-TIM-3 in PDAC.

#### 4.1.4. Anti-TIGIT

Activated CD4^+^ and CD8^+^ T cells, Tregs and NK cells express TIGIT. Ligation of TIGIT with its ligands CD155 and CD112, expressed on tumor and myeloid cells, promotes NK and T cell tolerance [76,77]. There is abundant expression of CD155 in tumor tissue (primarily tumor cells) of PDAC patients, with expression levels inversely correlated with TIL frequency and survival [78]. TIGIT competes with its opposing receptor CD226 for its ligands, the latter receptor enforcing anti-tumor responses through NK and T cell activation. Accordingly, it was shown that anti-TIGIT treatment selectively affects CD226^hi^CD8^+^ T cells in a pre-clinical setting [79]. Modified FOLFIRINOX treatment in mPDAC patients increased the proportion of CD226^hi^CD8^+^ T cells, implying that this chemotherapeutic combination may increase tumor sensitivity to anti-TIGIT treatment. A phase 1b/2 randomized trial is currently using anti-TIGIT combined with gemcitabine/nab-paclitaxel in mPDAC patients (ClinicalTrials.gov Identifier: NCT03193190).

#### 4.1.5. Anti-LAG-3

LAG-3 potentiates inhibitory signals in T cells upon TCR/MHC interaction. In addition, it is known for its synergism with PD-1. While anti-LAG-3 has shown little effect as monotherapy, dual anti-LAG-3/anti-PD-1 treatment exerted potent anti-tumor effects in mice with melanoma and colon carcinoma [80]. Consequently, a bispecific antibody targeting LAG-3 and PD-1 was developed and tested in a murine pancreatic tumor model [81]. Initial results demonstrated that this antibody targeted CD4^+^ T cells, increasing their effector functions by releasing cytolytic granzyme B and interferon (IFN)-γ, followed by complete suppression of tumor growth. Clinical trials have yet to determine its clinical value and none are currently ongoing for PDAC.

### 4.2. Agonistic Immune Stimulators

#### 4.2.1. MHC Class II Agonist

Eftilagimod alpha, or IMP321, is an MHC class II agonist that triggers maturation of APCs followed by activation of CD8^+^ T cells. To date, one phase 1 clinical trial has explored the safety profile of IMP321 combined with gemcitabine in treatment-naïve advanced PDAC patients (Table 3) [82]. A total of 18 patients were enrolled, of whom none reported serious adverse events. Overall, combination treatment with IMP321 and gemcitabine was well-tolerated. No significant differences were noted in pre- and post-treatment serum levels of monocytes (CD11b^+^CD14^+^), conventional DCs (CD11c^+^) and T cell subsets (CD4^+^ or CD8^+^). From the lack of immunological response, the investigators concluded that higher doses of IMP321 may be required for future clinical trials.

#### 4.2.2. OX40 Agonist

OX40-OX40L ligation enhances effector functions, memory formation and survival of CD4^+^ and CD8^+^ T cells [87]. Pre-clinical research in a PDAC mouse model demonstrated that OX40 agonists significantly improved survival compared to anti-PD-1 monotherapy [88]. Moreover, when the OX40 agonists and anti-PD-1 were combined the proportion of Tregs and exhausted T cells decreased, whereas memory CD4^+^ and CD8^+^ T cell numbers increased. While OX40 agonists have demonstrated their potent immune-stimulating capacities in other advanced cancer patients [89], clinical data for PDAC is lacking. A phase 1b/2 trial is currently recruiting in which an OX40 agonist will be combined with a toll-like receptor (TLR)-9 ligand for, among other things, patients with mPDAC (ClinicalTrials.gov Identifier: NCT04387071).

#### 4.2.3. CD137 (4-1BB) Agonist

CD137, or 4-1BB, is expressed on primed T and NK cells. CD137-CD137L ligation protects antigen-specific T cells from undergoing apoptosis and promotes their effector functions and differentiation into memory T cells [90]. Pre-clinical research, in which CD137 agonists were evaluated in murine PDAC models, provided promising results in terms of survival and immune stimulation [91,92,93]. Clinical reports on the use of CD137 agonists in PDAC patients are limited to a single phase 1 clinical trial, in which an adoptive T cell strategy was employed (discussed in “Adoptive Cell Therapies”) [94].

#### 4.2.4. ICOS Agonist

The ICOS pathway plays a dual role in cancer immunology [95,96]. Activation of this pathway has been shown to induce immunosuppression through CD4^+^ T cell subsets including Tregs. On the other hand, it can also unleash Th1 cell-mediated immunity and has demonstrated the ability to enhance the anti-tumor efficacy of GVAX [97,98]. Besides initial pre-clinical results in a PDAC model, in which combined co-stimulation with ICOS and CD137 has shown to increase the persistence of chimeric antigen receptor (CAR) T cells (CAR T cell therapy will be discussed in “Adoptive cell therapies”) [92], no clinical trials have yet been published.

#### 4.2.5. CD40 Agonist

CD40 is a co-stimulatory receptor found on APCs rather than on T cells. Its ligand, CD40L, is present primarily on activated CD4^+^ T cells [99]. CD40 ligation on DCs induces positive signaling that leads to their maturation and the release of IL-12, resulting in anti-tumor activity through T cell proliferation and differentiation towards a Th1 dominant state. Remarkably, one study in an in vivo PDAC model showed this efficacious anti-tumor activity to be directly mediated by CD40-expressing macrophages rather than T cells [83]. Since PDAC has a high macrophage content this triggered high interest in the clinical application of agonistic CD40 antibodies in PDAC. CD40 agonists, in combination with mesothelioma-lysate loaded DCs [100] or IL-15 [101], have demonstrated promising pre-clinical results in PDAC models. Beatty et al. [83,84] combined a CD40 agonist with gemcitabine in 22 chemotherapy-naïve advanced PDAC patients (Table 3). Results from this phase 1 trial revealed an mOS and mPFS of 8.5 and 5.2 months, respectively. While this treatment did not improve survival compared to gemcitabine/nab-paclitaxel, the immune system was activated, evidenced by the increase in inflammatory cytokines, a boost in B-cell expansion of co-stimulatory molecules and overall depletion of B cells. In their multi-cohort phase 1b trial, O’Hara et al. [85] utilized a similar regimen ± anti-PD-1 in synchronous and metachronous mPDAC patients (allowed prior chemoradiotherapy). They found encouraging initial mOS (12.7–20.1 months) and mPFS (10.8–12.5 months) outcomes, leading to the ongoing randomized phase 2 portion of the study.

#### 4.2.6. CD27 Agonist/Anti-CD70

Activation of the co-stimulatory receptor CD27 promotes survival and differentiation of T cells towards effector and memory subtypes [102]. Hence, an agonistic CD27 antibody was tested in patients with (non-pancreatic) solid malignancies, resulting in biological and clinical activity [103]. No trials are currently ongoing that employ a CD27 agonist in PDAC patients. The CD27 ligand CD70 is generally only expressed on activated immune cells as it is regulated by the presence of antigens. However, aberrant CD70 expression has been noted in tumor tissue of 25% of PDAC patients [104]. Importantly, although the CD27-CD70 axis is known for its immune stimulatory effects, CD70-expressing (pancreatic) cancer cells may also instigate immune tolerance by increasing the frequency of activated Tregs [105]. Since normal tissues have negligible expression, CD70 may present as a therapeutic target. Anti-CD70 antibodies have demonstrated potent anti-tumor activity in a murine PDAC model overexpressing CD70 [104] and good tolerability and preliminary anti-tumor activity in various CD70 positive advanced (non-pancreatic) solid cancers [106,107]. However, no clinical trials are currently recruiting CD70-overexpressing PDAC patients.

### 4.3. Cytokines

Immune stimulatory cytokines, such as IL-2, IL-15, GM-CSF and IFN-α, have been utilized as a subsidiary component in broader immunotherapy approaches in PDAC [57,108,109,110,111,112,113,114,115]. Solitary cytokine therapy had initial success in PDAC when used in the peri-operative phase [116,117]. However, no studies on this topic have been reported in the past decade.

### 4.4. Adjuvants

**Figure 3:**
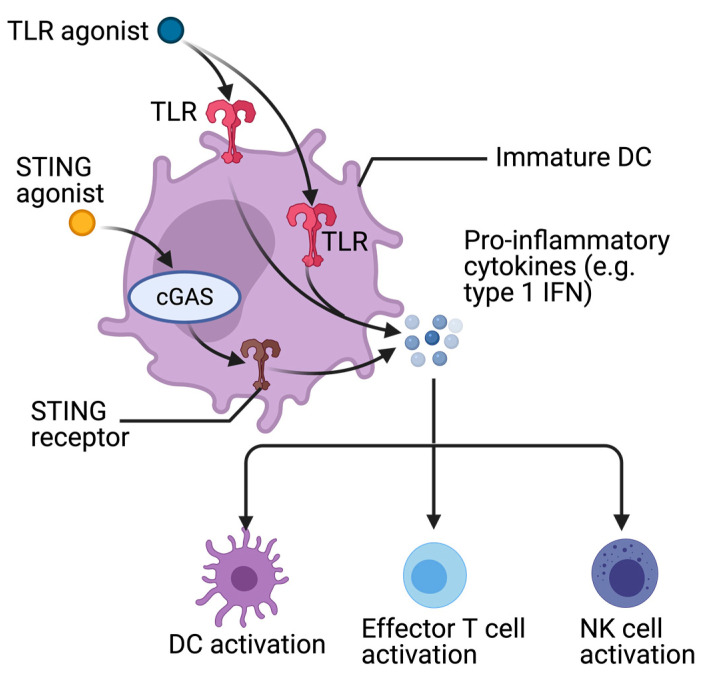
Adjuvants. Toll-like receptor (TLR) agonists bind onto transmembrane or intracellular TLRs and stimulator of interferon (IFN) genes (STING) agonists activate the intracellular cyclic GMP-AMP synthase (cGAS)-STING pathway in immature dendritic cells (DCs). Both agonists prompt release of pro-inflammatory cytokines, including type 1 IFN. These IFNs activate other DCs, effector T cells and natural killer (NK) cells.

In immunotherapy, adjuvants (Figure 3) usually refer to products that are added to vaccines in order to modulate or increase an immune response against the antigens contained within them [118]. However, these adjuvant compounds may also be used as monotherapy or as a supplement to other types of cancer treatment. They target the priming phase or the effector phase but can also be utilized as immune modulators to condition the microenvironment of both tumors and their draining lymph nodes in order to support both phases. Adjuvants bind onto pattern recognition receptors (PRRs), including toll-like receptors (TLRs), stimulator of IFN genes (STING) and NOD-like receptors (NLRs), which are present on epithelial (cancer) cells and innate immune cells, including DCs, and initiate immune responses against pathogens or tumor cells in secondary lymphoid organs [119]. Moreover, in tumors they may trigger release of chemokines and cytokines that can attract T cells and drive their effector response. Adjuvants can be delivered systemically, locally through vaccination or by peri- or intratumoral injection. A considerable advantage of intratumoral adjuvant administration over systemic approaches is that lower dosages will suffice, limiting severe toxicities [120,121]. Moreover, peritumoral administration has demonstrated superior anti-tumor efficacy in terms of DC and tumor-specific CD8^+^ T cell activation and long-lasting tumor protection in mice when compared to intravenous or intradermal administration [122].

#### 4.4.1. Toll-Like Receptor (TLR) Agonists

In humans, there are ten functioning TLRs, TLR-1 through TLR-10. Ligation of TLRs on innate immune cells initiates the release of pro-inflammatory cytokines like IL-12, IFN-α and TNF-α, prompting a positive feedback loop of DC maturation, and activation of effector T cells and NK cells (Figure 3), followed by a systemic anti-tumor effect [123,124]. However, TLR expression has also been observed in pancreatic tumor cells and, likely context-dependent, has been linked to both pro- and anti-tumor effects [125]. High cytoplasmic expression of various TLRs in tumor cells positively correlated with a favorable prognosis in PDAC patients [126,127,128]. Conversely, TLR ligation in pancreatic cancer cells has also been linked to tumorigenesis through inflammatory responses that stimulate their anti-apoptotic properties and angiogenesis [129,130,131]. Pre-clinical evidence in PDAC models demonstrated encouraging results of TLR agonist monotherapy [132,133,134] or combined with chemotherapy [135], local tumor eradication (i.e., radiotherapy or ablation) and/or other immunotherapies [136,137,138]. Initial clinical results in PDAC patients are also encouraging. Dalgleish et al. [86] published data of a randomized phase 2 trial in which they employed intradermally injected IMM-101, a TLR-2/1 agonist, in combination with gemcitabine (*n* = 75) and compared clinical efficacy to gemcitabine (*n* = 35) only in 110 LAPC and mPDAC patients (Table 3). A significant (*p* = 0.01) improvement in survival was noted for mPDAC patients in the IMM-101 + gemcitabine group (mOS 7 months) compared to gemcitabine monotherapy (mOS 4.4 months). Two clinical trials are currently investigating an intratumoral TLR-9 agonist for mPDAC, both in combination with anti-PD-1 and a local form of tumor eradication (ClinicalTrials.gov Identifier: NCT04612530 (PANFIRE-III trial [139]); NCT04050085).

#### 4.4.2. Stimulator of Interferon Genes (STING) Agonists

Cyclic GMP-AMP synthase (cGAS)-STING is an intracellular sensor pathway in innate immune cells that detects cytosolic double stranded DNA fragments [140]. Similar to TLR activation, stimulation of the cGAS-STING pathway results in the production of type 1 IFN and other pro-inflammatory cytokines, instigating activation of effector T cells and NK cells, and maturation of DCs (Figure 3). In pre-clinical PDAC mouse models, STING agonists have demonstrated potent anti-tumor efficacy by increasing cytotoxic T cell activity and decreasing Treg levels [141]. Moreover, STING activation augmented co-stimulatory receptor expression on DCs and converted immune-suppressive macrophages into immune-activating subtypes. Combined with other immunotherapies [138,142,143], or radiotherapy [144], STING agonists in PDAC murine models have demonstrated the ability to induce durable tumor regression and to improve survival. Nonetheless, two initial clinical trials (ClinicalTrials.gov Identifier: NCT03010176; NCT03172936) that employed respectively intratumoral and intravenous STING agonists in patients with solid cancers and lymphomas failed to demonstrate favorable results in terms of tumor regression [145]. The ongoing clinical trial ClinicalTrials.gov Identifier: NCT04144140 is administering intratumoral STING agonists in various tumor types.

#### 4.4.3. NOD-Like Receptor (NLR) Agonists

NLRs can cooperate with TLRs and are able to detect pathogen-associated molecular patterns (PAMPs) and danger or stress signals, thereby regulating inflammatory and apoptotic responses [146]. However, the therapeutic potential of targeting these pathways in PDAC has not yet been explored.

### 4.5. Oncolytic Viruses

**Figure 4:**
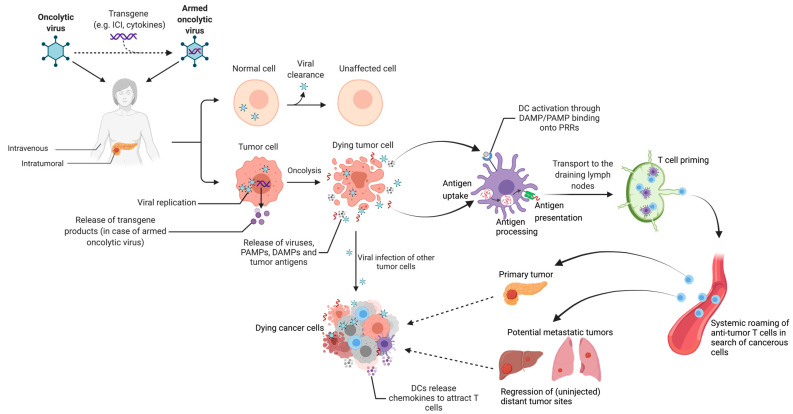
Oncolytic viral therapy. Oncolytic viruses may be equipped with a transgene (e.g., immune checkpoint inhibitor (ICI) or cytokine), creating an armed oncolytic virus. Oncolytic viruses can be administered intravenously or intratumorally and will infect both healthy cells and tumor cells. In the former, viruses are cleared, whereas in the latter, due to activation of oncogenic pathways and a defective interferon (IFN) response, oncolytic viruses thrive, leading to production of novel viral particles to a point where the cell lyses due to viral overload. Newly created oncolytic viruses, pathogen-associated molecular patterns (PAMPs), damage-associated molecular patterns (DAMPs) and tumor antigens are released into the microenvironment. New waves of released oncolytic viruses can then infect other tumor cells. Dendritic cells (DCs) take up, process and present the released antigens and upon activation by the released DAMPs and PAMPs via their pattern recognition receptors (PRRs), will transport them to the draining lymph nodes where T cell priming takes place. Primed effector T cells (Th1 CD4^+^, CD8^+^) subsequently provide systemic immune surveillance and will migrate towards the original tumor site (but also to distant, uninjected metastatic sites) via a chemokine gradient, often provided by activated DCs.

Oncolytic viral therapy utilizes the destructive capacity of a virus, specifically targeted at tumor cells whilst normal healthy cells remain unaffected (Figure 4). Activated oncogenic pathways and a defective IFN response (normally limiting viral replication), both specific to tumor cells, allow for selective replication of the virus, leading to production of abundant novel viral particles and eventual tumor cell lysis. The newly produced oncolytic viruses, virus-derived PAMPs, DAMPs and tumor antigens are released into the TME, infecting other tumor cells and activating DCs followed by T cell priming in the draining lymph nodes (i.e., in vivo vaccination). Subsequently, effector T cells are attracted to the tumor site via a gradient of chemokines such as CXCL9 and CXCL10 [147,148]. A major benefit of oncolytic viruses is their genetically modifiable genome, allowing for incorporation of immune modulator transgenes (“armed oncolytic viruses”), which are also released by infected tumor cells. Such immunological arming may be aimed at decreasing immune suppression in the TME and/or increasing immune activation.

In murine PDAC models, oncolytic viruses have demonstrated the capacity to reduce tumor burden and prolong survival by downregulating TAMs and increasing infiltration and function of Th1 CD4^+^ cells and CD8^+^ T cells [149,150]. A few clinical trials have utilized oncolytic viruses in PDAC (Table 4) [151,152,153,154,155]. Noonan et al. [154] reported data of their RCT, in which patients received intravenous pelareorep (an oncolytic reovirus) combined with carboplatin/paclitaxel (*n* = 36) or carboplatin/paclitaxel alone (*n* = 37). The primary outcome, mPFS, did not significantly differ between the treatment groups (4.9 vs. 5.2 months, *p* = 0.6). However, addition of pelareorep did result in demonstrable immune responses with increased systemic levels of pro-inflammatory cytokines, Th1 CD4^+^ and CD8^+^ T cells, yet also enhanced Treg numbers. Using the same oncovirus in combination with anti-PD-1 and chemotherapy, Mahalingam et al. [50] showed the generation of new T cell clones, recirculating systemically and transcriptional evidence of systemic immune activation, both associated with clinical benefit. This suggests the priming and mobilization of new T cell clones, providing systemic protection and possibly setting the stage for subsequent successful immune checkpoint blockade. Hirooka et al. [155] utilized intratumoral HF-10, a natural oncolytic HSV-1 virus, in combination with erlotinib (anti-EGFR antibody) and gemcitabine in 10 LAPC patients, achieving an mOS of 15.5 months. Multiple clinical trials incorporating an oncolytic virus in PDAC patients are currently recruiting (ClinicalTrials.gov Identifier: NCT02705196; NCT04637698; NCT03252808). Several other trials have been completed; however, results have not yet been published (ClinicalTrials.gov Identifier: NCT02045589; NCT02653313 [156]).

### 4.6. Adoptive Cell Therapies

**Figure 5:**
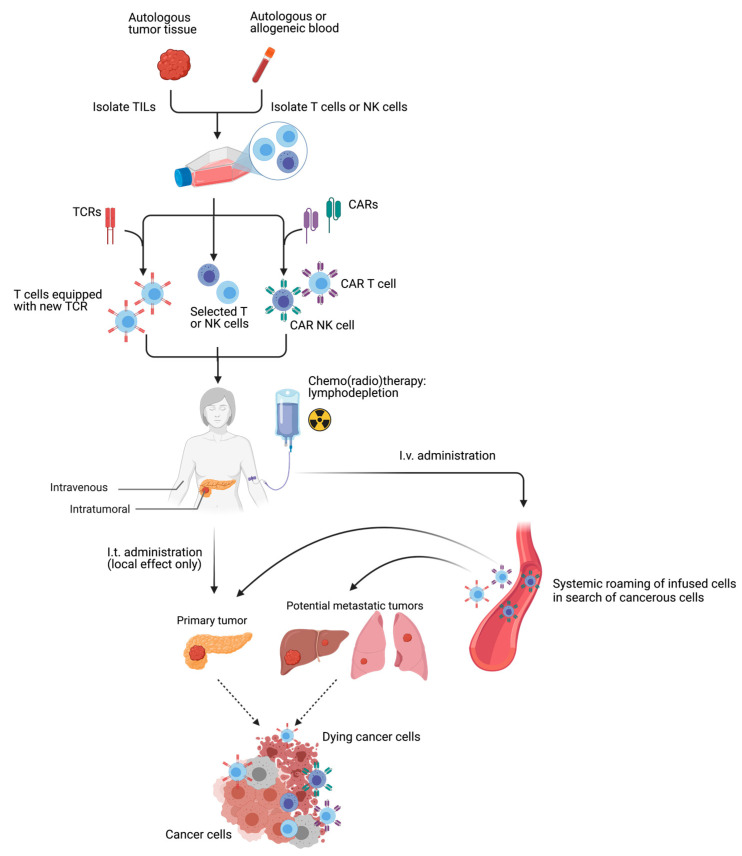
Adoptive cell therapy. Allogeneic (donor) or autologous (patient) blood can be used to isolate T cells or NK cells. Similarly, autologous tumor tissue can be collected to isolate tumor-infiltrating lymphocytes (TILs), generally T cells. These isolated cells are cultured in vitro and can be selected for expansion based on their anti-tumoral capacity or enhanced with T cell receptors (TCRs) or chimeric antigen receptors (CARs). Following in vitro manipulation and stimulation and, in case of systemic administration, subsequent to a lymphodepleting chemo(radio)therapy regimen, these cells are injected intratumorally (i.t.) or intravenously (i.v.) to (systemically) detect and kill cancer cells.

Adoptive cell therapies utilize autologous or allogeneic immune effector cells, such as T cells or NK cells, to eradicate cancer (Figure 5). After harvesting, either from the patient’s tumor or from blood, the immune effector cells are selected and their functionality may be improved in vitro—e.g., through genetic modification to express a chimeric antigen receptor (CAR) that targets a specific protein or a TCR to specifically recognize a peptide/MHC complex—followed by re-administration back into the patient. The application of CAR-engineered cells in solid tumors is still in an early developmental stage and requires optimization to achieve long-term persistence post-transfer and sufficient infiltration into the tumor fields.

A pre-clinical study in a murine PDAC model has yielded promising data after treatment with mesothelin-directed CAR T cells combined with armed oncolytic viruses expressing IL-2 and TNF-α [150]. Clinically (Table 5), CAR T cell therapy has not yet been widely explored for PDAC, but initial results, using mesothelin-directed (± CD137 and CD3ζ) CAR T cells, showed limited activity [94,157]. Aoki et al. [158] utilized Vγ9Vδ2 T cells, a subset of T cells that secrete Th1-related cytokines and have the capacity to exert potent cytotoxicity against tumor cells through MHC-unrestricted recognition of phosphoantigens. After surgical resection of the primary pancreatic tumor, patients (*n* = 40) received gemcitabine as adjuvant therapy, with (*n* = 23) or without (*n* = 17) Vγ9Vδ2 T cell infusion. This supplemental therapy was unable to prolong mPFS or mOS, but patients with >15% peripheral Vγ9Vδ2 T cells after two injections had a more favorable outcome. Conversely, Kumai et al. [159] recently demonstrated that αβ T cell therapy ± chemotherapy resulted in an mOS of 11.3 months from start of immunotherapy and 18.7 months from diagnosis, indicating a survival benefit compared to previous reports in a similar patient population receiving chemotherapy only (6.8–11.1 months from diagnosis) [4,5]. In addition, increased levels of CD8^+^ T cells were found in peripheral blood samples. Several clinical trials are currently ongoing to further explore CAR T cell potential in PDAC (ClinicalTrials.gov Identifier: NCT04037241; NCT03638193; NCT03323944) and TIL therapy (ClinicalTrials.gov Identifier: NCT03935893; NCT04426669). A trial employing NK cells combined with ablation will be discussed in ‘Immunotherapy + IRE’. Several NK cell therapy trials are recruiting (ClinicalTrials.gov Identifier: NCT03634501; NCT03093688) or have been completed without publication (ClinicalTrials.gov Identifier: NCT03008304).

### 4.7. Tumor Vaccines

**Figure 6:**
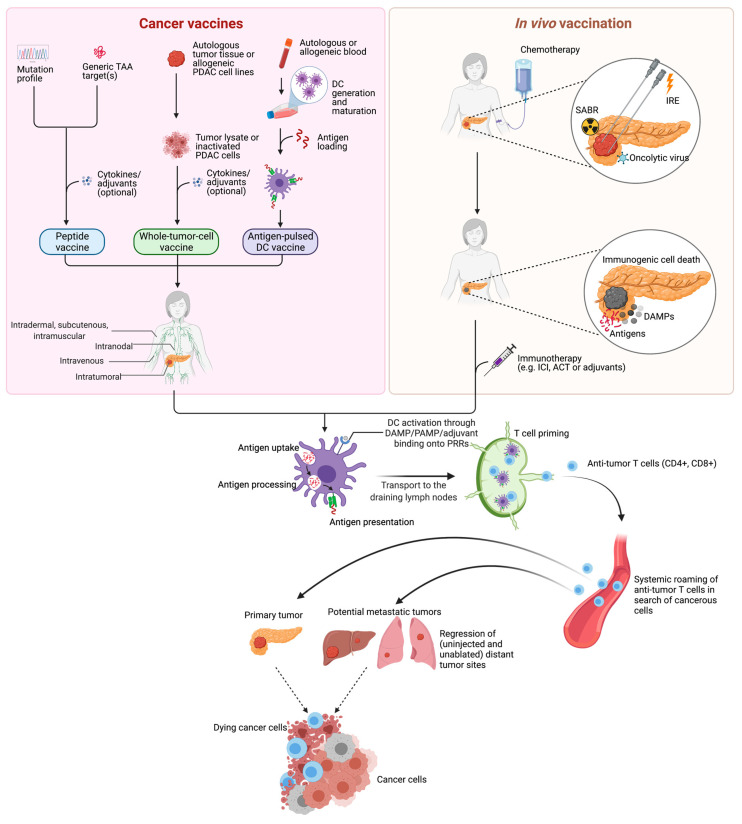
Cancer vaccines and in vivo vaccination following chemotherapy, ablation or oncolytic viral therapy. **Cancer vaccines:** Peptide-based vaccines can be produced on the basis of a patient’s mutational profile (i.e., KRAS (or other) neoantigens) or using (a combination of) generic tumor-associated antigens (TAAs). Whole-tumor-cell vaccines can be generated with autologous tumor lysate or using (a combination of) allogeneic PDAC cell lines. Peptide vaccines and whole-tumor-cell vaccines may be supplemented with cytokines, such as granulocyte macrophage colony-stimulating factor (GM-CSF), or adjuvants, which attracts and activates dendritic cells (DCs). For DC vaccines, autologous or allogeneic DCs are generated in vitro from patient or donor derived precursors in blood (e.g., monocytes), followed by antigen pulsing. Cancer vaccines may be administered intratumorally, intravenously, intranodally or subcutaneously, intradermally or intramuscularly. **In vivo vaccination:** Chemotherapy, oncolytic viruses and local ablative treatments (e.g., stereotactic ablative body radiotherapy (SABR) and irreversible electroporation (IRE)) have the capacity to initiate immunogenic cell death, thereby releasing damage-associated molecular patterns (DAMPs) and antigens, followed by alleviation of tumor-induced immune suppression and tumor-specific T cell priming. The immune response may be further leveraged by employing other immunotherapies such as immune checkpoint inhibitors (ICI), adoptive cell therapies (ACT) or adjuvants. **Systemic immune response:** With the exception of antigen-pulsed DC vaccines, all other forms of vaccination require in vivo antigen uptake of DCs. Activated DCs transport the antigens to the draining lymph nodes and establish antigen-specific priming and expansion of T cells. Primed effector anti-tumor T cells (Th1 CD4^+^ and CD8^+^ cytolytic T cells) roam the system in search of tumor cells and upon discovery may destroy these cancerous cells.

Tumor vaccines clinically pursued in the treatment of PDAC mainly include whole-tumor-cell, peptide-based and peptide-pulsed DC vaccines (Figure 6). They may contain one or several tumor-associated antigens (TAAs) or tumor-specific antigens (TSAs). TAAs are proteins overexpressed or aberrantly expressed in tumor cells but are also expressed to some extent on non-cancerous tissues, hence making them non-specific. TAA vaccines include, but are not limited to, those containing wilms tumor (WT)-1, kinesin family member (KIF)20A, VEGFR1/2, survivin, mucin (MUC)-1, mesothelin and human telomerase reverse transcriptase (hTERT) (Table 6) [160]. Mutated TSAs, or neoantigens, are expressed exclusively on tumor cells. Neoantigen vaccines are highly specific, immunogenic and avoid self-antigen-induced T cell tolerance [161]. However, their working mechanism relies on the mutational burden of a tumor, which is limited in PDAC [37]. In other words, these vaccines may not be able to kill (subgroups of) tumor cells lacking expression of that neoantigen [13]. Neoantigen targets, an example of which are kirsten rat sarcoma (KRAS) mutations, may be identified through mutational analyses. Vaccines can be administered intratumorally, intranodally, intradermally, intravenously, subcutaneously and intramuscularly [162].

#### 4.7.1. GVAX

Pancreas GVAX contains irradiated allogeneic pancreatic cancer cells, unable to grow, administered intradermally and comprising a plethora of PDAC antigens. In addition, the vaccine has been virally transduced with GM-CSF, an immune stimulatory cytokine with the ability to attract and activate conventional DCs (cDCs) [193], thus facilitating cross-priming of antigens contained within the constituent allogeneic cell lines. Thus far, results have demonstrated that GVAX, with or without concurrent treatments, is safe and well-tolerated and can induce antigen-specific T cell responses [57,108,109,110,111,112]. However, it has yet to show superior efficacy compared to current standard chemotherapy. In their phase 2b RCT of 169 mPDAC patients, Le et al. [110] showed no improved survival for patients receiving GVAX plus cyclophosphamide (Cy, aiming to eliminate Tregs) plus CRS-207 (i.e., a *Listeria Monocytogenes*-based bacterial vaccine expressing mesothelin) or CRS-207 only, compared to chemotherapy (mOS 3.7 months, 5.4 months and 4.6 months, respectively, *p >* 0.05). Additionally, Tsujikawa et al. [111] showed no benefit of supplemental anti-PD-1 when added to a GVAX regimen (GVAX + Cy + CRS-207) in 93 mPDAC patients, with mOS of 5.9 months versus 6.2 months. However, patients whose TME showed an increase in CD8^+^ T cells and a decrease in TAMs and MDSCs, had a better OS. The same group also published an RCT in which the value of GVAX + anti-CTLA-4 was explored in a maintenance setting where mPDAC patients had already received 4–6 doses of FOLFIRINOX. Patients (*n* = 82) were randomized to receive the experimental treatment (GVAX + anti-CTLA-4, *n* = 40) or continue standard FOLFIRINOX (*n* = 42). While dual immunotherapy did promote memory T cell differentiation and increased the M1 macrophage population, it proved significantly inferior in terms of survival compared to FOLFIRINOX (mOS 9.4 months vs. 14.7 months, ORR 6% vs. 14%, *p* = 0.019).

#### 4.7.2. WT-1

WT-1 plays a crucial role in tumor growth, invasion, angiogenesis and metastatic processes, with overexpression observed in approximately 75% of PDAC patients [194,195,196]. WT-1 peptide vaccines [163,164,165,166,167,168,169,170,171] and WT-1 peptide-pulsed DCs [165,166,167,168,169,171] have been utilized in combination with chemotherapy. Nishida et al. [169] presented data on the largest cohort, including 85 patients with recurrent, LAPC or mPDAC who were randomized to receive an intradermal WT-1 peptide vaccine plus gemcitabine (*n* = 42) or gemcitabine monotherapy (*n* = 43). No significant difference in overall survival was observed (mOS 9.6 months vs. 8.9 months (*p* = 0.4); mPFS 5.2 months vs. 3.3 months (*p* = 0.08)) was noted, but patients with positive a delayed-type hypersensitivity (DTH) had a substantially improved PFS (*p >* 0.001). On the other hand, Nagai et al. [171] employed a WT-1/MUC-1 peptide-pulsed DC vaccine combined with gemcitabine as adjuvant therapy in resected PDAC patients. Up to 40% of patients generated a WT-1-specific CD8^+^ T cell response. While they had no direct control group, the reported survival outcomes were favorable and encourage comparative studies, mPFS and mOS of 17.7 and 46.4 months from the first vaccination, respectively.

#### 4.7.3. KIF20A

KIF20A belongs to the superfamily of motor proteins which play an essential role in the trafficking of molecules during pancreatic cancer growth [197] and is highly expressed in >90% of PDAC patients [198]. Initial results have been published of KIF20A vaccines for PDAC [172,173,176,177]. Asahara et al. [172] utilized a subcutaneously injected KIF20A-66 protein vaccine in gemcitabine refractory, unresectable or recurrent metastatic PDAC patients (*n* = 29), resulting in a peptide-specific CD8^+^ effector T cell response and an mOS of 4.2 months, compared to 2.2 months after best supportive care (*p* = 0.047).

#### 4.7.4. VEGFR

Under physiological conditions, VEGF is essential for angiogenesis during embryonic growth, normal growth and tissue repair. However, in cancer this process has become deregulated due to abnormal expression of VEGF, resulting in anomalous blood vessel structures, thereby promoting tumor growth, invasion and spread, while hindering proper lymphocyte homing and extravasation [199]. VEGF and its receptors, VEGFR1/2, are co-expressed in 77–93% of PDAC patients and overexpression is linked to worse clinical prognosis [200,201]. A few articles have presented clinical data of VEGFR vaccines in PDAC [174,175,176,177]. Yamaue et al. [175] reported results of a phase 2/3 RCT in which 153 chemoradiation naïve LAPC and mPDAC patients were included and allocated either a subcutaneously injected VEGFR2 peptide vaccine combined with gemcitabine (*n* = 100) or a placebo with gemcitabine (*n* = 53). Results revealed a comparable mOS between the two treatment groups (8.4 and 8.5 months, *p* = 0.9, respectively). Similar results were presented by Miyazawa et al. [174]

#### 4.7.5. Survivin

Survivin is physiologically expressed during embryonic and fetal development and plays a critical role in cell cycle control and apoptosis. In PDAC it is re-expressed in about 80% of patients [202], with elevated expression associated with worse prognosis and treatment resistance [203]. Shima et al. [178,179] utilized a survivin 2B-vaccine (SVN-2B) in a phase 2 clinical trial with randomization design in which 83 advanced PDAC patients were included, all pre-treated with at least one line of chemotherapy. Patients were allocated to receive SVN-2B + IFN-α (*n* = 30), SVN-2B only (*n* = 34) or a placebo (*n* = 19). While survivin-specific CD8^+^ T cells were increased, mOS outcomes were similar between the three arms (3.4 months, 3.2 months and 3.6 months, respectively).

#### 4.7.6. MUC-1

MUC-1 is expressed at intermediate levels by pancreatic tissue, orchestrating cell signaling and differentiation functions. However, atypical expression is seen in >60% of PDAC patients and correlates with tumor size and dysplasia, suggesting a pivotal role in tumor progression [204]. Rong et al. [180] employed a MUC-1-pulsed DC vaccine in 7 advanced PDAC patients previously treated with chemotherapy and surgery, all of whom had aberrant MUC-1 expression. ELISPOT assays showed an increase in IFN-γ and granzyme B secreting peripheral blood mononuclear cells (PBMCs) in 29% of patients, but a clinical response was not observed (ORR 0%, DCR 0%).

#### 4.7.7. Mesothelin

Mesothelin is minimally expressed in several healthy tissues and is almost always (~100%) highly expressed in PDAC at its invading edges, but its exact functions remain unknown [205]. It is often co-expressed with CA-125 (MUC-16). Binding of CA-125 with secreted mesothelin enhances tumor cell motility and invasion [206]. In chemotherapy refractory PDAC patients, CRS-207 (the mesothelin-expressing bacterial vaccine) increased mesothelin-specific CD8^+^ T cells in 60% of patients and resulted in an mOS of 7 months [181].

#### 4.7.8. hTERT

Telomeric ends of DNA strings become progressively shortened with cellular replication, eventually leading to senescence and death. Pancreatic cancer cells are able to reverse this process by re-activating telomerase enzymes, which can elongate shortened telomeres, hereby inducing replicative immortality [207]. Middleton et al. [115] published a phase 3 RCT, including LAPC and mPDAC patients (*n* = 1082) of whom a subset was treated with an intradermal telomerase peptide vaccine (GV1001). Patients were randomized to receive chemotherapy (*n* = 358), sequential chemo-immunotherapy (GV1001/GM-CSF) (*n* = 350) or concurrent chemo-immunotherapy (GV1001/GM-CSF) (*n* = 354). Adding GV001 did not improve survival; the mOS outcomes were 7.9 months, 6.9 months and 8.4 months, respectively (*p* = 0.11). For both chemo-immunotherapy groups an immunological response was measured; in the sequential group 12% of patients were DTH positive and 31% demonstrated GV1001-specific T cell proliferation, whereas in the concurrent group 20% of patients were DTH+ and 15% demonstrated GV1001-specific T cell proliferation.

#### 4.7.9. Neoantigen: Mutant KRAS

The KRAS protein is part of the RAS/Mitogen-Activated Protein Kinase (MAPK) signaling pathway, involved in cell proliferation and differentiation. In more than 90% of PDAC patients the *KRAS* proto-oncogene is mutated, its expression being associated with overall worse prognosis and treatment insensitivity [208]. In clinical trials, a mutant KRAS vaccine has been utilized through an Epstein Barr Virus-transformed lymphoblastoid cell line (CLC) [183] or combined with adjuvant GM-CSF [113,114] in PDAC patients with a confirmed *KRAS* mutation. Mutant KRAS vaccines combined with gemcitabine as adjuvant therapy have demonstrated encouraging initial results in resected PDAC patients. Palmer et al. [114] published data from a phase 1/2 clinical trial, in which 32 PDAC patients with resected primary tumors received an intradermal injection of a seven-peptide vaccine covering most known mutations of *KRAS* (TG01), co-administered with recombinant GM-CSF (TG01/GM-CSF), and combined with gemcitabine. Their survival outcomes (mOS 33.1–34.3 months; mPFS 13.9–19.5 months) compared favorably with current literature reporting survival after resection followed by adjuvant gemcitabine (mOS 17–27 months) [209,210,211,212]. In addition, patients presented with enhanced levels of immune activation; 62–95% of patients were skin-tested and showed a positive DTH reaction, 74–92% of patients had peptide-specific T cells.

#### 4.7.10. Neoantigen: Other

Neoantigen vaccines are fairly novel but have already seen some preliminary clinical success in multiple solid tumors, even poorly immunogenic glioblastoma, in terms of safety, immunogenicity and feasibility [213,214,215,216]. A pre-clinical study using a murine PDAC model demonstrated that a combined neoantigen vaccine and STING adjuvant regimen resulted in transient tumor regression [142]. Moreover, adding anti-PD-1 and an OX40 agonist enhanced these results. Clinically, there is only a proof-of-antigen discovery feasibility study done in three resected PDAC patients [184]. A multitude of clinical trials are currently recruiting PDAC patients for neoantigen vaccine treatments (e.g., ClinicalTrials.gov Identifier: NCT03558945, NCT03953235, NCT03956056).

#### 4.7.11. Tumor-Based/Multiple Antigen Vaccines

Tumor-based vaccines and multi-antigen vaccines, with or without DCs as delivery vector, have been utilized clinically for PDAC [185,186,187,188,189,190,191,192]. As first-line treatment, a personalized peptide vaccine was combined with gemcitabine in 21 LAPC and mPDAC patients, resulting in an mOS of 9 months and mPFS of 7 months [185], similar to gemcitabine/nab-paclitaxel alone (8.5 months). Impressive results were demonstrated when alpha-galactosyl (α-Gal)-expressing tumor lysate-pulsed DCs were combined with cytokine-induced killer cells (CIK) [189]. Pre-treated LAPC and mPDAC patients (*n* = 14) achieved an mOS of 24.7 months from diagnosis and CD8^+^ T cells, CD45^+^RO^+^ T cells and CD56^+^ NK cell levels were increased. DTH was positive in 86% of patients and correlated with prognosis (*p* > 0.01). Mehrotra et al. [192] combined a peptide-pulsed hTERT DC vaccine, carcinoembryonic antigen (CEA) and survivin with an intramuscular TLR-3 agonist (poly-ICLC) in pre-treated LAPC and mPDAC patients, resulting in an mOS and mPFS of 7.7 and 3 months from immunotherapy treatment. This treatment combination induced measurable and tumor-specific T cell populations. Other studies confirm the ability of these multi-antigen vaccines to induce an anti-tumor immune response [185,186,187,188,190,191].

### 4.8. Immunotherapy and Local Ablation

Focal ablation techniques have, in addition to their tumor destructive capacity, demonstrated immune potentiating abilities by inducing immunogenic cell death and temporarily lifting immune-suppressive conditions [11,217,218]. In effect, this form of tumor destruction may prompt in vivo vaccination against patient-specific pancreatic tumor cells (Figure 6). Combining ablation with immunotherapy might act synergistically to elicit an anti-tumor immune response. Of the available ablation techniques, thus far only irreversible electroporation (IRE) and stereotactic ablative body radiotherapy (SABR) have been combined with immunotherapy in PDAC patients (Table 7).

#### 4.8.1. Immunotherapy + IRE

IRE has demonstrated the ability to temporarily lift immune-suppressive barriers and to elicit an anti-tumor immune response in PDAC patients [224,225]. Consequently, the feasibility of combined IRE with immunotherapy, coined as ‘electroimmunotherapy’ [226], was examined in pre-clinical immunocompetent PDAC murine models, resulting in significant tumor regression, increased CD8^+^ T cell infiltration and improved survival [137,227,228,229]. In a clinical setting, percutaneous IRE has been combined with allogeneic NK cell therapy [219]. Compared to IRE alone, IRE + NK cells resulted in a modestly improved mOS in patients with LAPC (12.2 vs. 13.6 months, *p* = 0.033, *n* = 35) and mPDAC (9.1 vs. 10.2 months, *p* = 0.037, *n* = 32), as well as improved mPFS in LAPC (9.1 months vs. 7.9 months, *p* = 0.043). Furthermore, patients receiving NK cell therapy demonstrated significantly increased systemic levels of total and CD8^+^ T cells, NK cells and pro-inflammatory cytokines. Recently, an RCT was published in which IRE + allogeneic Vγ9Vδ2 T cells (*n* = 30) was compared to IRE alone (*n* = 32) in 62 LAPC patients, of whom 49 had previously received chemotherapy [220]. Clinical outcomes for IRE + Vγ9Vδ2 T cells were superior compared to IRE alone (mOS 14.5 months vs. 11 months, *p* = 0.01; mPFS 11 months vs. 8 months, *p* = 0.03). Interestingly, patients receiving multiple T cell infusions survived significantly longer compared to patients receiving a single infusion (mOS 17 months vs. 13.5 months, *p >* 0.05). O’Neill et al. [221] provided initial results of a phase 1b trial in which a combination of surgical IRE and systemic anti-PD-1 was administered to ten LAPC patients. All patients were stable after first line chemotherapy (8 FOLFIRINOX, 2 gemcitabine/nab-paclitaxel). The mOS was 18 months and mPFS was 6.3 months, thus providing a solid base for their subsequent, currently ongoing phase 2 clinical trial. Finally, the PANFIRE-III trial (NCT04612530) is currently evaluating safety and efficacy of IRE + systemic anti-PD-1 ± an intratumoral TLR-9 agonist in mPDAC patients with at least stable disease after pre-treatment with a minimum of eight FOLFIRINOX cycles [139].

#### 4.8.2. Immunotherapy + SABR

Pre-clinical treatment strategies in murine PDAC models combining SABR and intratumoral anti-CD40 or intratumoral IL-12 microspheres presented promising results. IFN-γ production increased and CD8^+^ T cells were activated, followed by significant tumor regression and formation of long-term immunologic memory [230,231]. Clinically, Xie et al. [223] examined combination treatment with SABR and immune checkpoint inhibitors in 59 pre-treated mPDAC patients. Interestingly, SABR + anti-PD-L1 (mOS of 3.3–9 months) resulted in superior survival compared to SABR + anti-PD-L1 + anti-CTLA-4 (mOS 2.1–4.2 months). An increase in CD3^+^ and CD8^+^ T cells was observed independent of clinical response. While this study was limited due to an absent control arm, SABR + anti-PD-L1 may confer a minor survival benefit in previously treated mPDAC patients. In another clinical trial, a multimodal treatment strategy including chemotherapy, SABR, nelfinavir (anti-retroviral drug, i.e., a radiosensitizer), an anti-CA-125 antibody ± pancreatic resection in LAPC patients (*n* = 11) resulted in an mOS and mPFS of 13 and 8.6 months, respectively [222]. Of the five patients tested, 40% developed CA-125 specific CD8^+^ T cells. Overall, this treatment approach was safe and feasible, but did not result in a major clinical benefit. Multiple trials are ongoing that combine SABR with various immunotherapies in PDAC (e.g., ClinicalTrials.gov Identifier: NCT03563248; NCT04390399; NCT04327986; NCT04098432).

## 5. Conclusions

Results from clinical trials incorporating immunotherapy strategies have been disappointing in PDAC. The limited efficacy, although of multifactorial causation, can largely be attributed to the highly immunosuppressive TME. In order to overcome this major obstacle, current immunotherapies such as oncolytic viruses, cancer vaccines and adoptive (CAR) T or NK cell therapies should be improved and optimized. In addition, novel immunotherapy targets identified in a pre-clinical context for which limited or no clinical data in PDAC exists should be further explored to determine their potential. These include novel immune checkpoint inhibitors, stimulatory agonists and adjuvants. Equally, the complex interplay of immunosuppressive mechanisms likely requires rational multimodal treatment approaches, targeting several immune evasion mechanisms to establish a durable anti-tumor response. In order to achieve this response, antigen presentation should be improved, anti-tumor immune cells activated, checkpoint-induced inhibition removed and tumor-site infiltration of cytotoxic immune cells increased. To make such optimized treatment choices, careful dissection of immune evasion mechanisms at play in the human PDAC TME and its draining lymph nodes is required. The motive for employing combination therapies is reinforced by the demonstrable potential of various immunotherapies to elicit a tumor-specific immune response. Moreover, therapies with the ability to provoke immunogenic cell death (e.g., chemotherapy and ablative treatment) may work synergistically with immunotherapy. For these strategies, optimal timing, dosage and choice of combination therapies may emerge as the primary challenge. Ultimately, the aim is to develop a (personalized) multimodal treatment strategy which will lift immunosuppressive barriers, effectively kill all tumor cells and create long-lasting protective immune memory.

## Figures and Tables

**Table 1:** An overview of co-inhibitory (red) and co-stimulatory (green) receptor pairs.

Antigen Presenting Cell or Tumor Cell (Ligand)	T Cell (Receptor)
MHC class I or II (antigen loaded)	TCR (signal 1), LAG-3
PD-L1 (B7-H1), PD-L2 (B7-DC)	PD-1
Gal-9	TIM-3
CD155, CD112	TIGIT
CD80 (B7-1), CD86 (B7-2)	CD28 (signal 2), CTLA-4
ICOSL	ICOS
CD137L	CD137 (4-1BB)
OX40L	OX40
CD70	CD27
**Antigen Presenting Cell or Tumor Cell (Receptor)**	**T Cell (Ligand)**
CD40	CD40L

**Table 2:** Clinical studies on immune checkpoint inhibitors.

Authors	Year	Phase	Immunotherapy	Combined with	No. of Patients	Patient Population	mPFS (Months)	mOS (Months)
Anti-PD-1/ PD-L1								
Brahmer [44]	2012	1	Anti-PD-L1	-	14	Previously treated LAPC/mPDAC	NR	NR
Weiss [47]	2017	1b	Anti-PD-1	+ chemo	11	Previously treated mPDAC	NR	8 (x)
Weiss [48]	2018	1b/2	Anti-PD-1	+ gem/nab-pac	17	mPDAC, 11/17 chemotherapy-naïve	9.1	15 (x)
Doi [51]	2019	1	Anti-PD-1	+ anti-CCR4	15	Previously treated mPDAC	1.8	6.5 (x)
Hong [52]	2019	1b/2	Anti-PD-L1	+ TKI	49	Previously treated LAPC/mPDAC	1.7	4.2 (t)
O’Reilly [46]	2019	2	Anti-PD-L1 Anti-CTLA-4	Randomization: (1) − (2) + anti-CTLA-4	65	Previously treated mPDAC	NR	NR
Bockorny [49]	2020	2a	Anti-PD-1	Two cohorts: (1) + anti-CXCR4 (2) + anti-CXCR4 + chemo	59	Previously treated mPDAC	NR	(1) 3.3 (t) (2) 7.8 (t)
Marabelle [45]	2020	2	Anti-PD-1	-	22	Previously treated MSI-H LAPC/mPDAC	NR	NR
Mahalingam [50]	2020	1b	Anti-PD-1	+ oncolytic virus + chemo	11	Previously treated LAPC/mPDAC	2	3.1 (x)
Anti-CTLA-4								
Royal [56]	2010	2	Anti-CTLA-4		27	Previously treated LAPC/mPDAC	NR	NR
Le [57]	2013	1b	Anti-CTLA-4	Randomization: (1) − (2) + GVAX	30	Previously treated LAPC/mPDAC		(1) 3.6 (t) (2) 5.7 (t)
Aglietta [58]	2014	1	Anti-CTLA-4	+ gem	34	chemotherapy naïve mPDAC	NR	7.4 (x)
Mohindra [59]	2015	1b	Anti-CTLA-4	+ gem	13	Previously treated LAPC/mPDAC	NR	NR
Kalyan [60]	2016	1b	Anti-CTLA-4	+ gem	16	Previously treated LAPC/mPDAC	2.5	8.3 (x)
Kamath [61]	2020	1b	Anti-CTLA-4	+ gem	21	Previously treated LAPC/mPDAC	2.8	6.9 (x)

NR = not reported; gem = gemcitabine; nab-pac = nab-paclitaxel; CCR4 = C-C chemokine receptor 4; CXCR4 = C-X-C Motif Chemokine Receptor 4; TKI = tyrosine kinase inhibitor; GVAX = GM-CSF transduced allogeneic cell line-based vaccine; LAPC = locally advanced pancreatic cancer; mPDAC = metastatic pancreatic ductal adenocarcinoma; mPFS = median progression-free survival; mOS = median overall survival; t = from treatment/registration; d = from diagnosis; x = unknown whether from diagnosis or treatment/registration.

**Table 3:** Clinical studies on immune stimulatory agonists and adjuvants.

Authors	Year	Phase	Immunotherapy	Combined with	No. of Patients	Patient Population	mPFS (Months)	mOS (Months)
MHC-II agonist								
Wang-Gillam [82]	2013	1	MHC-II agonist	(1) + gem (2) gem alone (control)	18	Treatment-naïve LAPC/mPDAC	(1) 2–5.3 (2) 10.2	(1) 5.6–6.4 (x) (2) 16.7 (x)
CD40 agonist								
Beatty [83,84]	2011, 2013	1	CD40 agonist	+ gem	22	Chemotherapy-naïve LAPC/mPDAC	5.2	8.4 (t)
O’Hara [85]	2021	1b	CD40 agonist	+ gem ± anti-PD1	24	Recurrent or synchronous mPDAC	10.8–12.5	12.7–20.1 (t)
TLR agonist								
Dalgleish [86]	2016	2	TLR2/1 agonist (i.t.)	(1) + gem (2) gem alx one (control)	110	Chemotherapy-naïve LAPC/mPDAC	NR	mPDAC: (1) 7 (t) (2) 4.4 (t)

NR = not reported; i.t. = intratumoral; gem = gemcitabine; LAPC = locally advanced pancreatic cancer; mPDAC = metastatic pancreatic ductal adenocarcinoma; mPFS = median progression-free survival; mOS = median overall survival; t = from treatment/registration; d = from diagnosis; x = unknown whether from diagnosis or treatment/registration.

**Table 4:** Clinical studies on oncolytic viruses.

Authors	Year	Phase	Immunotherapy	Admin. Site	Combined with	No. of Patients	Patient Population	mPFS (Months)	mOS (Months)
Oncolytic viruses									
Nakao [151], Kasuya [152]	2011, 2014	1	HF10 (HSV)	i.t.	-	6	Unresectable (intraoperative) PDAC	NR	6.2 (x)
Aguilar [153]	2015	1	Adenoviral vector expressing HSV-tk gene	i.t.	+ anti-herpetic prodrug	24	As adjuvant of: (1) Surgical resection (2) LAPC: CRT	(1) NR (2) 5.8	(1) NR (2) 12 (t)
Noonan [154]	2016	2	Pelareorep	i.v.	(1) + carb/pac (2) carb/pac alone	73	Treatment-naïve mPDAC	(1) 4.9 (2) 5.2	(1) 7.3 (t) (2) 8 (t)
Hirooka [155]	2018	1	HF10 (HSV)	i.t.	+ gem + erlotinib	10	Chemotherapy-naïve LAPC	6.3	15.5 (t)

NR = not reported; HSV = herpes simplex virus; i.v. = intravenous; i.t. = intratumoral; gem = gemcitabine; carb/pac = carboplatin/paclitaxel; LAPC = locally advanced pancreatic cancer; mPDAC = metastatic pancreatic ductal adenocarcinoma; mPFS = median progression-free survival; mOS = median overall survival; t = from treatment/registration; d = from diagnosis; x = unknown whether from diagnosis or treatment/registration.

**Table 5:** Clinical studies on T cell therapy.

Authors	Year	Phase	Immunotherapy	Combined with	No. of Patients	Patient Population	mPFS (Months)	mOS (Months)
T cell therapy								
Aoki [158]	2017	1	Vγ9Vδ2 T cells	(1) + gem (2) gem alone	40	Resected PDAC	NR	NR
Beatty [157]	2018	1	CAR T cells: mesothelin-directed	-	6	Previously treated mPDAC	NR	NR
Haas [94]	2019	1	CAR T cells: Transduced with mesothelin, 4-1BB and CD3ζ	-	5	Previously treated mPDAC	NR	NR
Kumai [159]	2021	NR	αβ T cells	± chemo	77	Previously treated LAPC/mPDAC	NR	11.3 (t) 18.7 (d)

NR = not reported; CAR = chimeric antigen receptor; gem = gemcitabine; chemo = chemotherapy; LAPC = locally advanced pancreatic cancer; mPDAC = metastatic pancreatic ductal adenocarcinoma; mPFS = median progression-free survival; mOS = median overall survival; t = from treatment/registration; d = from diagnosis.

**Table 6:** Clinical studies on pancreatic tumor vaccines.

Authors	Year	Phase	Immunotherapy	Type of Vaccine	Admin. Site	Combined with	No. of Patients	Patient Population	mPFS (Months)	mOS (Months)
GVAX										
Lutz [108]	2011	2	GVAX (+ GM-CSF)	Whole-tumor-cell	i.d.	+ resection + CRT	60	Resected PDAC	17.3	24.8 (t)
Le [109]	2015	2	GVAX (+ GM-CSF)	Whole-tumor-cell	i.d.	(1) + Cy + CRS-207 (2) + Cy	90	Pre-treated mPDAC	NR	(1) 6.1 (t) (2) 3.9 (t)
Le [110]	2019	2b	GVAX (+ GM-CSF)	Whole-tumor-cell	i.d.	(1) + Cy + CRS-207 (2) + Cy (3) chemo only	169	Pre-treated mPDAC	(1) 2.3 (2) 2.1 (3) 2.1	(1) 3.7 (t) (2) 5.4 (t) (3) 4.6 (t)
Tsujikawa [111]	2020	2	GVAX (+ GM-CSF)	Whole-tumor-cell	i.d.	(1) + Cy + CRS-207 + anti-PD-1 (2) + Cy + CRS-207	93	Pre-treated mPDAC	(1) 2.2 (2) 2.2	(1) 5.9 (t) (2) 6.1 (t)
Wu [112]	2020	2	GVAX (+ GM-CSF)	Whole-tumor-cell	i.d.	(1) + anti-CTLA-4 (2) FFX only (continuation)	82	Pre-treated mPDAC (4–6 doses FFX)	(1) 2.4 (2) 5.6	(1) 9.4 (t) (2) 14.7 (t)
WT-1										
Kaida [163]	2011	1	WT-1 vaccine	Peptide	i.d.	+ gem	9	Gem-naïve LAPC/mPDAC	NR	8.2 (t)
Nishida [164]	2014	1	WT-1 vaccine	Peptide	i.d.	+ gem	32	Treatment-naïve LAPC/mPDAC and treated recurrent disease	4.2	8.1 (t)
Koido [165]	2014	1	WT-1 vaccine	DC	i.d.	+ gem	10	mPDAC: Treatment-naïve newly diagnosed or recurrence after resection	NR	NR
Tsukinaga [166]	2015	NR	WT-1 vaccine	DC	i.d.	+ gem	7	Treatment-naïve mPDAC	6.8	10.7 (t)
Mayanagi [167]	2015	1	WT-1 vaccine	DC	i.d.	+ gem	10	Treatment-naïve LAPC/mPDAC	NR	8 (t)
Yanagisawa [168]	2018	1	WT-1 vaccine	DC	i.d.	+ chemo	8	Resected, chemo-naïve PDAC	NR	NR
Nishida [169]	2018	2	WT-1 vaccine	Peptide	i.d.	(1) + gem (2) gem alone	85	Treatment-naïve LAPC, mPDAC or recurrence after resection	(1) 5.2 (2) 3.3	(1) 9.6 (t) (2) 8.9 (t)
Hanada [170]	2020	NR	WT-1 vaccine	DC	i.d.	-	6	Pre-treated (CRT) PDAC: Resected, mPDAC, recurrent	19.9	59 (x)
Nagai [171]	2020	1/2a	WT-1/MUC-1 vaccine	DC	i.d.	+ gem	10	Resected PDAC	17.7	46.4 (t)
KIF20A										
Asahara [172]	2013	1/2	KIF20A vaccine	Peptide	s.c.	(1) -(2) no treatment (BSC)	110	Chemo-refractory, LAPC/mPDAC or recurrence after resection	(1) 1.8 (2) NR	(1) 4.7 (x) (2) 2.1–2.7 (x)
Suzuki [173]	2014	1	KIF20A vaccine	Peptide	s.c.	+ gem	9	Pre-treated LAPC/mPDAC	NR	5.7 (t) 18 (d)
VEGFR										
Miyazawa [174]	2010	1	VEGFR2 vaccine	Peptide	s.c.	+ gem	18	LAPC/mPDAC, 17% treatment-naïve	3.9	7.7 (t)
Yamaue [175]	2015	2/3	VEGFR2 vaccine	Peptide	s.c.	(1) + gem (2) gem only	153	Treatment-naïve LAPC/mPDAC	(1) 3.7 (2) 3.8	(1) 8.4 (t) (2) 8.5 (t)
KIF20A + VEGFR										
Suzuki [176]	2017	2	KIF20A + VEGFR1/2 vaccine	Peptide	s.c.	+ gem	68	Chemo-naïve LAPC/mPDAC	4.7–5.2	9–10 (t)
Miyazawa [177]	2017	2	KIF20A + VEGFR1/2 vaccine	Peptide	s.c.	+ gem	30	Resected PDAC	15.8	NR
Survivin										
Kameshima [178]	2013	NR	Survivin vaccine	Peptide	s.c.	+ IFA, IFN-α	6	LAPC/mPDAC/recurrence, unknown prior treatments	NR	NR
Shima [179]	2019	2	Survivin vaccine	Peptide	s.c.	(1) + IFA, IFN-β (2) + IFA(3) placebo only	83	Pre-treated LAPC/mPDAC	(1) 2.2 (3) 2.3	(1) 3.4 (t) (2) 3.2 (t) (3) 3.6 (t)
MUC-1										
Rong [180]	2012	1	MUC-1 vaccine	DC	i.d.	-	6	Pre-treated LAPC/mPDAC/recurrence	NR	NR
Mesothelin										
Le [181]	2012	1	Mesothelin-expressing *Lm* vaccine	*Lm*	i.v.	-	9	Treatment-refractory PDAC	NR	7 (x)
hTERT										
Middleton [115]	2014	1	Telomerase vaccine	Peptide	i.d.	(1) chemo only (2) + chemo + GM-CSF (sequential) (3) + chemo + GM-CSF (concurrent)	1062	Treatment-naïve LAPC/mPDAC	(1) 6.4 (2) 4.5 (3) 6.6	(1) 7.9 (t) (2) 6.9 (t) (3) 8.4 (t)
Neoantigen:mutant KRAS										
Wedén [113]	2011	1/2	KRAS vaccine	Peptide	i.d.	+ GM-CSF	23	Resected PDAC	NR	27.5 (t)
Abou-Alfa [182]	2011			Peptide	i.d.	+ GM-CSF	24	Resected PDAC	8.6	20.3 (t)
Kubuschok [183]	2012	1	KRAS vaccine	LCL	s.c.	-	7	mPDAC (recurrent or newly diagnosed)	3.1	4.5 (t)
Palmer [114]	2020	1/2	KRAS vaccine	Peptide	i.d.	+ GM-CSF + gem	32	Resected PDAC	13.9–19.5	33.1–34.2 (t)
Neoantigen:other										
Bassani-Sternberg [184]	2019	1b	Neoantigens	DC	s.c.	+ chemo + anti-PD-1 + aspirin	3	Resected PDAC	NR	NR
Tumor-based										
Yanagimoto [185]	2010	2	Personalized vaccine	Peptide	s.c.	+ gem	21	Treatment-naïve LAPC/mPDAC	7	9 (t)
Bauer [186]	2011	1	Tumor lysate vaccine	DC—Whole-tumor-cell	i.d.	+ gem	12	Recurrence after resection	NR	10.5 (dr)
Kimura [187]	2012	NS	Personalized and/or tumor lysate vaccine	DC	i.t.	+ chemo ± LAK cell therapy	49	Chemo-refractory LAPC/mPDAC	NR	11.8 (t)
Yutani [188]	2013	2	Personalized vaccine	Peptide	s.c.	+ chemo	41	Chemo-refractory mPDAC, newly diagnosed and recurrence after resection	NR	7.9 (t)
Qiu [189]	2013	1	Tumor lysate expressing α-Gal	DC	i.d.	+ CIK cell therapy	14	Pre-treated LAPC/mPDAC	NR	24.7 (d)
Lin [190]	2015	NS	Pancreatic cancer stem cell lysate	Whole-tumor-cell	s.c.	-	90	Pre-treated stage II PDAC, LAPC, mPDAC	NR	NR
Zhang [191]	2016	R	Various tumor antigens (unspecified)	DC	i.v.	(1) + CIK (2) no further treatment after baseline therapy	150	Pre-treated PDAC	NR	(1) 4.4 (t) (2) 3.8 (t)
Mehrotra [192]	2017	1	hTERT, CEA, survivin vaccine	DC	i.d.	+ TLR-3 agonist (i.m.)	12	Pre-treated LAPC/mPDAC	3	7.7 (t)

NR = not reported; R = retrospective; i.d. = intradermal; i.v. = intravenous; i.m. = intramuscular; i.t. = intratumoral; s.c. = subcutaneous; α-Gal = alpha-galectin; TLR = toll-like receptor; Cy = cyclophosphamide; CRS-207 = mesothelin-expressing *Lm* vaccine; *Lm* = *Listeria monocytogenes*; DC = dendritic cell, LAK = lymphokine-activated killer; CIK = cytokine-induced killer; FFX = FOLFIRINOX; gem = gemcitabine; chemo = chemotherapy; BSC = best supportive care; IFA = incomplete Freund’s adjuvant; LAPC = locally advanced pancreatic cancer; mPDAC = metastatic pancreatic ductal adenocarcinoma; mPFS = median progression-free survival; mOS = median overall survival; t = from treatment/registration; d = from diagnosis; dr = from diagnosis of recurrent disease; x = unknown whether from diagnosis or treatment/registration.

**Table 7:** Clinical studies combining immunotherapy with ablative therapies.

Authors	Year	Phase	Immunotherapy	Ablative Therapy	Approach	Treatment	No. of Patients	Patient Population	mPFS (Months)	mOS (Months)
IT + IRE										
Lin [219]	2017	NR	NK cell therapy	IRE	Perc.	(1) IRE only (2) IRE + NK cells	67	LAPC/mPDAC, 73% pre-treated with CRT	(1) 4.8–7.9 (2) 5.3–9.1	(1) 9.1–12.2 (t) (2) 10.2–13.6 (t)
Lin [220]	2020	NR	Vγ9Vδ2 T cell therapy	IRE	Perc.	(1) IRE only (2) IRE + T cells	62	LAPC, 79% pre-treated with chemo	(1) 8 (2) 11	(1) 11 (t) (2) 14.5 (t)
O’Neill [221]	2020	1b	Anti-PD-1	IRE	Open	IRE + anti-PD-1	10	LAPC, pre-treated with chemo	6.3	18 (x)
IT + SABR										
Lin [222]	2019	1/2	Anti-CA-125	SABR	-	SABR + chemo + nelfinavir + anti-CA-125 ± resection	11	LAPC/mPDAC, treatment-naïve	8.6	13 (t)
Xie [223]	2020	1	Anti-PD-L1 Anti-CTLA-4	SABR	-	(1) SABR + anti-PD-L1 (2) SABR + anti-PD-L1 + anti-CTLAA-4	59	LAPC/mPDAC, pre-treated chemo	(1) 1.7–2.5 (2) 0.9–2.3	(1) 3.3–9 (t) (2) 2.1–4.2 (t)

NR = not reported; IT = immunotherapy; IRE = irreversible electroporation; SABR = stereotactic ablative body radiotherapy; perc = percutaneous; chemo = chemotherapy; LAPC = locally advanced pancreatic cancer; mPDAC = metastatic pancreatic ductal adenocarcinoma; mPFS = median progression-free survival; mOS = median overall survival; t = from treatment/registration; d = from diagnosis; x = unknown whether from diagnosis or treatment/registration.

## References

[1] McGuigan A., Kelly P., Turkington R., Jones C., Coleman H.G., McCain R.S. (2018). Pancreatic cancer: A review of clinical diagnosis, epidemiology, treatment and outcomes. World J. Gastroenterol..

[2] Bükki J. (2014). Pancreatic Adenocarcinoma. N. Engl. J. Med..

[3] Bengtsson A., Andersson R., Ansari D. (2020). The actual 5-year survivors of pancreatic ductal adenocarcinoma based on real-world data. Sci. Rep..

[4] Conroy T., Desseigne F., Ychou M., Bouché O., Guimbaud R., Bécouarn Y., Adenis A., Raoul J.-L., Gourgou-Bourgade S., De La Fouchardière C. (2011). FOLFIRINOX versus Gemcitabine for Metastatic Pancreatic Cancer. N. Engl. J. Med..

[5] Von Hoff D.D., Ervin T., Arena F.P., Chiorean E.G., Infante J., Moore M., Seay T., Tjulandin S.A., Ma W.W., Saleh M.N. (2013). Increased Survival in Pancreatic Cancer with nab-Paclitaxel plus Gemcitabine. N. Engl. J. Med..

[6] Riquelme E., Maitra A., McAllister F. (2018). Immunotherapy for Pancreatic Cancer: More than Just a Gut Feeling. Cancer Discov..

[7] Pihlak R., Weaver J.M.J., Valle J.W., McNamara M.G. (2018). Advances in Molecular Profiling and Categorisation of Pancreatic Adenocarcinoma and the Implications for Therapy. Cancers.

[8] Huber M., Brehm C.U., Gress T.M., Buchholz M., Alhamwe B.A., Von Strandmann E.P., Slater E.P., Bartsch J.W., Bauer C., Lauth M. (2020). The Immune Microenvironment in Pancreatic Cancer. Int. J. Mol. Sci..

[9] Chen J., Xiao-Zhong G., Qi X.-S. (2017). Clinical Outcomes of Specific Immunotherapy in Advanced Pancreatic Cancer: A Systematic Review and Meta-Analysis. J. Immunol. Res..

[10] Galluzzi L., Humeau J., Buqué A., Zitvogel L., Kroemer G. (2020). Immunostimulation with chemotherapy in the era of immune checkpoint inhibitors. Nat. Rev. Clin. Oncol..

[11] Geboers B., Ruarus A.H., Nieuwenhuizen S., Puijk R.S., Scheffer H.J., De Gruijl T.D., Meijerink M.R. (2019). Needle-guided ablation of locally advanced pancreatic cancer: Cytoreduction or immunomodulation by in vivo vaccination?. Chin. Clin. Oncol..

[12] Karamitopoulou E. (2019). Tumour microenvironment of pancreatic cancer: Immune landscape is dictated by molecular and histopathological features. Br. J. Cancer.

[13] Das M., Zhou X., Liu Y., Das A., Vincent B.G., Li J., Liu R., Huang L. (2020). Tumor neoantigen heterogeneity impacts bystander immune inhibition of pancreatic cancer growth. Transl. Oncol..

[14] Martinez-Bosch N., Vinaixa J., Navarro P. (2018). Immune Evasion in Pancreatic Cancer: From Mechanisms to Therapy. Cancers.

[15] Gong R., Ren H. (2020). Targeting chemokines/chemokine receptors: A promising strategy for enhancing the immunotherapy of pancreatic ductal adenocarcinoma. Signal Transduct. Target. Ther..

[16] Li M., Bharadwaj U., Zhang R., Zhang S., Mu H., Fisher W.E., Brunicardi F.C., Chen C., Yao Q. (2008). Mesothelin is a malignant factor and therapeutic vaccine target for pancreatic cancer. Mol. Cancer Ther..

[17] Hiraoka N., Onozato K., Kosuge T., Hirohashi S. (2006). Prevalence of FOXP3+ Regulatory T Cells Increases During the Progression of Pancreatic Ductal Adenocarcinoma and Its Premalignant Lesions. Clin. Cancer Res..

[18] Gabitass R.F., Annels N.E., Stocken D.D., Pandha H.A., Middleton G. (2011). Elevated myeloid-derived suppressor cells in pancreatic, esophageal and gastric cancer are an independent prognostic factor and are associated with significant elevation of the Th2 cytokine interleukin-13. Cancer Immunol. Immunother..

[19] Sideras K., Braat H., Kwekkeboom J., van Eijck C., Peppelenbosch M., Sleijfer S., Bruno M. (2013). Role of the immune system in pancreatic cancer progression and immune modulating treatment strategies. Cancer Treat. Rev..

[20] Lin J.H., Huffman A.P., Wattenberg M.M., Walter D., Carpenter E.L., Feldser D.M., Beatty G.L., Furth E.E., Vonderheide R.H. (2020). Type 1 conventional dendritic cells are systemically dysregulated early in pancreatic carcinogenesis. J. Exp. Med..

[21] Yamamoto T., Yanagimoto H., Satoi S., Toyokawa H., Yamao J., Kim S., Terakawa N., Takahashi K., Kwon A.-H. (2012). Circulating Myeloid Dendritic Cells as Prognostic Factors in Patients with Pancreatic Cancer Who Have Undergone Surgical Resection. J. Surg. Res..

[22] Hirooka S., Yanagimoto H., Satoi S., Yamamoto T., Toyokawa H., Yamaki S., Yui R., Inoue K., Michiura T., Kwon A.-H. (2011). The role of circulating dendritic cells in patients with unresectable pancreatic cancer. Anticancer. Res..

[23] Tjomsland V., Sandström P., Spångeus A., Messmer D., Emilsson J., Falkmer U., Falkmer S., Magnusson K.-E., Borch K., Larsson M. (2010). Pancreatic adenocarcinoma exerts systemic effects on the peripheral blood myeloid and plasmacytoid dendritic cells: An indicator of disease severity?. BMC Cancer.

[24] Yamamoto K., Venida A., Yano J., Biancur D.E., Kakiuchi M., Gupta S., Sohn A.S.W., Mukhopadhyay S., Lin E.Y., Parker S. (2020). Autophagy promotes immune evasion of pancreatic cancer by degrading MHC-I. Nat. Cell Biol..

[25] Bowers J., Bailey S.R., Rubinstein M.P., Paulos C.M., Camp E.R. (2019). Genomics meets immunity in pancreatic cancer: Current research and future directions for pancreatic adenocarcinoma immunotherapy. Oncol. Rev..

[26] Provenzano P., Cuevas C., Chang A., Goel V.K., Von Hoff D.D., Hingorani S.R. (2012). Enzymatic Targeting of the Stroma Ablates Physical Barriers to Treatment of Pancreatic Ductal Adenocarcinoma. Cancer Cell.

[27] Haqq J., Howells L.M., Garcea G., Metcalfe M.S., Steward W.P., Dennison A. (2014). Pancreatic stellate cells and pancreas cancer: Current perspectives and future strategies. Eur. J. Cancer.

[28] Whatcott C.J., Diep C.H., Jiang P., Watanabe A., LoBello J., Sima C., Hostetter G., Shepard H.M., Von Hoff D.D., Han H. (2015). Desmoplasia in Primary Tumors and Metastatic Lesions of Pancreatic Cancer. Clin. Cancer Res..

[29] Schizas D., Charalampakis N., Kole C., Economopoulou P., Koustas E., Gkotsis E., Ziogas D., Psyrri A., Karamouzis M.V. (2020). Immunotherapy for pancreatic cancer: A 2020 update. Cancer Treat. Rev..

[30] Pardoll D.M. (2012). The blockade of immune checkpoints in cancer immunotherapy. Nat. Rev. Cancer.

[31] He X., Xu C. (2020). Immune checkpoint signaling and cancer immunotherapy. Cell Res..

[32] Jung K.H., LoRusso P., Burris H., Gordon M., Bang Y.-J., Hellmann M.D., Cervantes A., de Olza M.O., Marabelle A., Hodi F.S. (2019). Phase I Study of the Indoleamine 2,3-Dioxygenase 1 (IDO1) Inhibitor Navoximod (GDC-0919) Administered with PD-L1 Inhibitor (Atezolizumab) in Advanced Solid Tumors. Clin. Cancer Res..

[33] Kamphorst A.O., Wieland A., Nasti T., Yang S., Zhang R., Barber D.L., Konieczny B.T., Daugherty C.Z., Koenig L., Yu K. (2017). Rescue of exhausted CD8 T cells by PD-1–targeted therapies is CD28-dependent. Science.

[34] Hui E., Cheung J., Zhu J., Su X., Taylor M.J., Wallweber H.A., Sasmal D.K., Huang J., Kim J.M., Mellman I. (2017). T cell costimulatory receptor CD28 is a primary target for PD-1–mediated inhibition. Science.

[35] Han Y., Liu D., Li L. (2020). PD-1/PD-L1 pathway: Current researches in cancer. Am. J. Cancer Res..

[36] Lin D.Y.-W., Tanaka Y., Iwasaki M., Gittis A.G., Su H.-P., Mikami B., Okazaki T., Honjo T., Minato N., Garboczi D.N. (2008). The PD-1/PD-L1 complex resembles the antigen-binding Fv domains of antibodies and T cell receptors. Proc. Natl. Acad. Sci. USA.

[37] Yarchoan M., Albacker L.A., Hopkins A.C., Montesion M., Murugesan K., Vithayathil T.T., Zaidi N., Azad N.S., Laheru D.A., Frampton G.M. (2019). PD-L1 expression and tumor mutational burden are independent biomarkers in most cancers. JCI Insight.

[38] Nomi T., Sho M., Akahori T., Hamada K., Kubo A., Kanehiro H., Nakamura S., Enomoto K., Yagita H., Azuma M. (2007). Clinical Significance and Therapeutic Potential of the Programmed Death-1 Ligand/Programmed Death-1 Pathway in Human Pancreatic Cancer. Clin. Cancer Res..

[39] Wang L., Ma Q., Chen X., Guo K., Li J., Zhang M. (2010). Clinical Significance of B7-H1 and B7-1 Expressions in Pancreatic Carcinoma. World J. Surg..

[40] Gao H.-L., Liu L., Qi Z.-H., Xu H.-X., Wang W.-Q., Wu C.-T., Zhang S.-R., Xu J.-Z., Ni Q.-X., Yu X.-J. (2018). The clinicopathological and prognostic significance of PD-L1 expression in pancreatic cancer: A meta-analysis. Hepatobiliary Pancreat. Dis. Int..

[41] Shindo Y., Hazama S., Suzuki N., Iguchi H., Uesugi K., Tanaka H., Aruga A., Hatori T., Ishizaki H., Umeda Y. (2017). Predictive biomarkers for the efficacy of peptide vaccine treatment: Based on the results of a phase II study on advanced pancreatic cancer. J. Exp. Clin. Cancer Res..

[42] Hodi F.S., Sileni V.C., Gonzalez R., Grob J.-J., Rutkowski P., Cowey C.L., Lao C.D., Schadendorf D., Wagstaff J., Dummer R. (2018). Nivolumab plus ipilimumab or nivolumab alone versus ipilimumab alone in advanced melanoma (CheckMate 067): 4-year outcomes of a multicentre, randomised, phase 3 trial. Lancet Oncol..

[43] Motzer R.J., Escudier B., McDermott D.F., George S., Hammers H.J., Srinivas S., Tykodi S.S., Sosman J.A., Procopio G., Plimack E.R. (2015). Nivolumab versus Everolimus in Advanced Renal-Cell Carcinoma. N. Engl. J. Med..

[44] Brahmer J.R., Tykodi S.S., Chow L.Q., Hwu W.-J., Topalian S.L., Hwu P., Drake C.G., Camacho L.H., Kauh J., Odunsi K. (2012). Safety and Activity of Anti–PD-L1 Antibody in Patients with Advanced Cancer. N. Engl. J. Med..

[45] Marabelle A., Le D.T., Ascierto P.A., Di Giacomo A.M., De Jesus-Acosta A., Delord J.-P., Geva R., Gottfried M., Penel N., Hansen A. (2020). Efficacy of Pembrolizumab in Patients With Noncolorectal High Microsatellite Instability/Mismatch Repair–Deficient Cancer: Results From the Phase II KEYNOTE-158 Study. J. Clin. Oncol..

[46] O’Reilly E.M., Oh D.-Y., Dhani N., Renouf D.J., Lee M.A., Sun W., Fisher G., Hezel A., Chang S.-C., Vlahovic G. (2019). Durvalumab With or Without Tremelimumab for Patients With Metastatic Pancreatic Ductal Adenocarcinoma. JAMA Oncol..

[47] Weiss G.J., Waypa J., Blaydorn L., Coats J., McGahey K., Sangal A., Niu J., A Lynch C., Farley J.H., Khemka V. (2017). A phase Ib study of pembrolizumab plus chemotherapy in patients with advanced cancer (PembroPlus). Br. J. Cancer.

[48] Weiss G.J., Blaydorn L., Beck J., Bornemann-Kolatzki K., Urnovitz H., Schütz E., Khemka V. (2017). Phase Ib/II study of gemcitabine, nab-paclitaxel, and pembrolizumab in metastatic pancreatic adenocarcinoma. Investig. New Drugs.

[49] Bockorny B., Semenisty V., Macarulla T., Borazanci E., Wolpin B.M., Stemmer S.M., Golan T., Geva R., Borad M.J., Pedersen K.S. (2020). BL-8040, a CXCR4 antagonist, in combination with pembrolizumab and chemotherapy for pancreatic cancer: The COMBAT trial. Nat. Med..

[50] Mahalingam D., Wilkinson G.A., Eng K., Fields P., Raber P., Moseley J.L., Cheetham K., Coffey M., Nuovo G., Kalinski P. (2019). Pembrolizumab in Combination with the Oncolytic Virus Pelareorep and Chemotherapy in Patients with Advanced Pancreatic Adenocarcinoma: A Phase Ib Study. Clin. Cancer Res..

[51] Doi T., Muro K., Ishii H., Kato T., Tsushima T., Takenoyama M., Oizumi S., Gemmoto K., Suna H., Enokitani K. (2019). A Phase I Study of the Anti-CC Chemokine Receptor 4 Antibody, Mogamulizumab, in Combination with Nivolumab in Patients with Advanced or Metastatic Solid Tumors. Clin. Cancer Res..

[52] Hong D., Rasco D., Veeder M., Luke J.J., Chandler J., Balmanoukian A., George T., Munster P., Berlin J.D., Gutierrez M. (2019). A Phase 1b/2 Study of the Bruton Tyrosine Kinase Inhibitor Ibrutinib and the PD-L1 Inhibitor Durvalumab in Patients with Pretreated Solid Tumors. Oncology.

[53] Zitvogel L., Galluzzi L., Smyth M.J., Kroemer G. (2013). Mechanism of Action of Conventional and Targeted Anticancer Therapies: Reinstating Immunosurveillance. Immunity.

[54] Cubas R., Moskalenko M., Cheung J., Yang M., McNamara E., Xiong H., Hoves S., Ries C.H., Kim J., Gould S. (2018). Chemotherapy Combines Effectively with Anti–PD-L1 Treatment and Can Augment Antitumor Responses. J. Immunol..

[55] Michelakos T., Cai L., Villani V., Sabbatino F., Kontos F., Castillo C.F.-D., Yamada T., Neyaz A., Taylor M.S., Deshpande V. (2020). Tumor Microenvironment Immune Response in Pancreatic Ductal Adenocarcinoma Patients Treated With Neoadjuvant Therapy. J. Natl. Cancer Inst..

[56] Royal R.E., Levy C., Turner K., Mathur A., Hughes M., Kammula U.S., Sherry R.M., Topalian S.L., Yang J.C., Lowy I. (2010). Phase 2 Trial of Single Agent Ipilimumab (Anti-CTLA-4) for Locally Advanced or Metastatic Pancreatic Adenocarcinoma. J. Immunother..

[57] Le D.T., Lutz E., Uram J.N., Sugar E.A., Onners B., Solt S., Zheng L., Diaz L., Donehower R.C., Jaffee E. (2013). Evaluation of Ipilimumab in Combination With Allogeneic Pancreatic Tumor Cells Transfected With a GM-CSF Gene in Previously Treated Pancreatic Cancer. J. Immunother..

[58] Aglietta M., Barone C., Sawyer M.B., Moore M.J., Miller W.H., Bagalà C., Colombi F., Cagnazzo C., Gioeni L., Wang E. (2014). A phase I dose escalation trial of tremelimumab (CP-675,206) in combination with gemcitabine in chemotherapy-naive patients with metastatic pancreatic cancer. Ann. Oncol..

[59] Mohindra N.A., Kircher S.M., Nimeiri H.S., Benson A.B., Rademaker A., Alonso E., Blatner N., Khazaie K., Mulcahy M.F. (2015). Results of the phase Ib study of ipilimumab and gemcitabine for advanced pancreas cancer. J. Clin. Oncol..

[60] Kalyan A., Kircher S.M., Mohindra N.A., Nimeiri H.S., Maurer V., Rademaker A., Benson A.B., Mulcahy M.F. (2016). Ipilimumab and gemcitabine for advanced pancreas cancer: A phase Ib study. J. Clin. Oncol..

[61] Kamath S.D., Kalyan A., Kircher S., Nimeiri H., Fought A.J., Benson A., Mulcahy M. (2019). Ipilimumab and Gemcitabine for Advanced Pancreatic Cancer: A Phase Ib Study. Oncology.

[62] Wei S.C., Duffy C.R., Allison J.P. (2018). Fundamental Mechanisms of Immune Checkpoint Blockade Therapy. Cancer Discov..

[63] Sansom D. (2000). CD28, CTLA-4 and their ligands: Who does what and to whom?. Immunology.

[64] Farren M., Mace T.A., Geyer S., Mikhail S., Wu C., Ciombor K.K., Tahiri S., Ahn D., Noonan A., A Villalonacalero M. (2015). Systemic Immune Activity Predicts Overall Survival in Treatment-Naïve Patients with Metastatic Pancreatic Cancer. Clin. Cancer Res..

[65] Ribas A. (2012). Tumor Immunotherapy Directed at PD-1. N. Engl. J. Med..

[66] Vargas F.A., Furness A.J., Litchfield K., Joshi K., Rosenthal R., Ghorani E., Solomon I., Lesko M.H., Ruef N., Roddie C. (2018). Fc Effector Function Contributes to the Activity of Human Anti-CTLA-4 Antibodies. Cancer Cell.

[67] Scherpereel A., Mazieres J., Greillier L., Lantuejoul S., Dô P., Bylicki O., Monnet I., Corre R., Audigier-Valette C., Locatelli-Sanchez M. (2019). Nivolumab or nivolumab plus ipilimumab in patients with relapsed malignant pleural mesothelioma (IFCT-1501 MAPS2): A multicentre, open-label, randomised, non-comparative, phase 2 trial. Lancet Oncol..

[68] Planchard D., Reinmuth N., Orlov S., Fischer J., Sugawara S., Mandziuk S., Marquez-Medina D., Novello S., Takeda Y., Soo R. (2020). ARCTIC: Durvalumab with or without tremelimumab as third-line or later treatment of metastatic non-small-cell lung cancer. Ann. Oncol..

[69] Acharya N., Sabatos-Peyton C., Anderson A.C. (2020). Tim-3 finds its place in the cancer immunotherapy landscape. J. Immunother. Cancer.

[70] Du W., Yang M., Turner A., Xu C., Ferris R.L., Huang J., Kane L.P., Lu B. (2017). TIM-3 as a Target for Cancer Immunotherapy and Mechanisms of Action. Int. J. Mol. Sci..

[71] Peng P.-J., Li Y., Sun S. (2017). On the significance of Tim-3 expression in pancreatic cancer. Saudi J. Biol. Sci..

[72] Wolf Y., Anderson A.C., Kuchroo V.K. (2019). TIM3 comes of age as an inhibitory receptor. Nat. Rev. Immunol..

[73] Cebrián M.J.G., Bauden M., Andersson R., Holdenrieder S., Ansari D. (2016). Paradoxical Role of HMGB1 in Pancreatic Cancer: Tumor Suppressor or Tumor Promoter?. Anticancer. Res..

[74] Gebauer F., Wicklein D., Horst J., Sundermann P., Maar H., Streichert T., Tachezy M., Izbicki J.R., Bockhorn M., Schumacher U. (2014). Carcinoembryonic Antigen-Related Cell Adhesion Molecules (CEACAM) 1, 5 and 6 as Biomarkers in Pancreatic Cancer. PLoS ONE.

[75] Seifert A.M., Reiche C., Heiduk M., Tannert A., Meinecke A.-C., Baier S., von Renesse J., Kahlert C., Distler M., Welsch T. (2020). Detection of pancreatic ductal adenocarcinoma with galectin-9 serum levels. Oncogene.

[76] Pauken K.E., Wherry E.J. (2014). TIGIT and CD226: Tipping the Balance between Costimulatory and Coinhibitory Molecules to Augment the Cancer Immunotherapy Toolkit. Cancer Cell.

[77] Johnston R.J., Comps-Agrar L., Hackney J., Yu X., Huseni M., Yang Y., Park S., Javinal V., Chiu H., Irving B. (2014). The Immunoreceptor TIGIT Regulates Antitumor and Antiviral CD8 + T Cell Effector Function. Cancer Cell.

[78] Nishiwada S., Sho M., Yasuda S., Shimada K., Yamato I., Akahori T., Kinoshita S., Nagai M., Konishi N., Nakajima Y. (2015). Clinical significance of CD155 expression in human pancreatic cancer. Anticancer. Res..

[79] Jin H.-S., Ko M., Choi D.-S., Kim J.H., Lee D.-H., Kang S.-H., Kim I., Lee H.J., Choi E.K., Kim K.-P. (2020). CD226hiCD8+ T Cells Are a Prerequisite for Anti-TIGIT Immunotherapy. Cancer Immunol. Res..

[80] Woo S.-R., Turnis M.E., Goldberg M.V., Bankoti J., Selby M., Nirschl C., Bettini M.L., Gravano D.M., Vogel P., Liu C.L. (2011). Immune Inhibitory Molecules LAG-3 and PD-1 Synergistically Regulate T-cell Function to Promote Tumoral Immune Escape. Cancer Res..

[81] Cebada J., Flores A., Bandala C., Lizaliturri-Flores I., Villa-Ruano N., Perez-Santos M. (2020). Bispecific anti-PD-1/LAG-3 antibodies for treatment of advanced or metastatic solid tumors: A patent evaluation of US2018326054. Expert Opin. Ther. Patents.

[82] Wang-Gillam A., Plambeck-Suess S., Goedegebuure P., Simon P.O., Mitchem J., Hornick J.R., Sorscher S., Picus J., Suresh R., Lockhart A.C. (2012). A phase I study of IMP321 and gemcitabine as the front-line therapy in patients with advanced pancreatic adenocarcinoma. Investig. New Drugs.

[83] Beatty G., Chiorean E.G., Fishman M.P., Saboury B., Teitelbaum U.R., Sun W., Huhn R.D., Song W., Li D., Sharp L.L. (2011). CD40 Agonists Alter Tumor Stroma and Show Efficacy against Pancreatic Carcinoma in Mice and Humans. Science.

[84] Beatty G.L., Torigian D.A., Chiorean E.G., Saboury B., Brothers A., Alavi A., Troxel A., Sun W., Teitelbaum U.R., Vonderheide R.H. (2013). A Phase I Study of an Agonist CD40 Monoclonal Antibody (CP-870,893) in Combination with Gemcitabine in Patients with Advanced Pancreatic Ductal Adenocarcinoma. Clin. Cancer Res..

[85] O’Hara M.H., O’Reilly E.M., Varadhachary G., A Wolff R., A Wainberg Z., Ko A.H., Fisher G., Rahma O., Lyman J.P., Cabanski C.R. (2021). CD40 agonistic monoclonal antibody APX005M (sotigalimab) and chemotherapy, with or without nivolumab, for the treatment of metastatic pancreatic adenocarcinoma: An open-label, multicentre, phase 1b study. Lancet Oncol..

[86] Dalgleish A.G., Stebbing J., Adamson D.J., Arif S.S., Bidoli P., Chang D., Cheeseman S., Diaz-Beveridge R., Fernandez-Martos C., Glynne-Jones R. (2016). Randomised, open-label, phase II study of gemcitabine with and without IMM-101 for advanced pancreatic cancer. Br. J. Cancer.

[87] Lu X. (2020). OX40 and OX40L interaction in cancer. Curr. Med. Chem..

[88] Ma Y., Li J., Wang H., Chiu Y., Kingsley C.V., Fry D., Delaney S.N., Wei S.C., Zhang J., Maitra A. (2020). Combination of PD-1 Inhibitor and OX40 Agonist Induces Tumor Rejection and Immune Memory in Mouse Models of Pancreatic Cancer. Gastroenterology.

[89] Curti B.D., Kovacsovics-Bankowski M., Morris N., Walker E., Chisholm L., Floyd K., Walker J., Gonzalez I., Meeuwsen T., Fox B.A. (2013). OX40 Is a Potent Immune-Stimulating Target in Late-Stage Cancer Patients. Cancer Res..

[90] Etxeberria I., Glez-Vaz J., Teijeira A., Melero I. (2019). New emerging targets in cancer immunotherapy: CD137/4-1BB costimulatory axis. ESMO Open.

[91] Muth S.T., Saung M.T., Blair A.B., Henderson M.G., Thomas D.L., Zheng L. (2020). CD137 agonist-based combination immunotherapy enhances activated, effector memory T cells and prolongs survival in pancreatic adenocarcinoma. Cancer Lett..

[92] Guedan S., Posey J.A., Shaw C., Wing A., Da T., Patel P.R., McGettigan S., Casado-Medrano V., Kawalekar O.U., Uribe-Herranz M. (2018). Enhancing CAR T cell persistence through ICOS and 4-1BB costimulation. JCI Insight.

[93] Sakellariou-Thompson D., Forget M.-A., Creasy C., Bernard V., Zhao L., Kim Y.U., Hurd M.W., Uraoka N., Parra E.R., Kang Y. (2017). 4-1BB Agonist Focuses CD8+ Tumor-Infiltrating T-Cell Growth into a Distinct Repertoire Capable of Tumor Recognition in Pancreatic Cancer. Clin. Cancer Res..

[94] Haas A.R., Tanyi J.L., O’Hara M.H., Gladney W.L., Lacey S.F., Torigian D.A., Soulen M.C., Tian L., McGarvey M., Nelson A.M. (2019). Phase I Study of Lentiviral-Transduced Chimeric Antigen Receptor-Modified T Cells Recognizing Mesothelin in Advanced Solid Cancers. Mol. Ther..

[95] Solinas C., Gu-Trantien C., Willard-Gallo K. (2020). The rationale behind targeting the ICOS-ICOS ligand costimulatory pathway in cancer immunotherapy. ESMO Open.

[96] Amatore F., Gorvel L., Olive D. (2018). Inducible Co-Stimulator (ICOS) as a potential therapeutic target for anti-cancer therapy. Expert Opin. Ther. Targets.

[97] Jong J.M.V.D.-D., Santegoets S.J., Van De Ven P.M., Versluis J., Verheul H., De Gruijl T.D., Gerritsen W.R., Eertwegh A.J.M.V.D. (2015). Improved efficacy of mitoxantrone in patients with castration-resistant prostate cancer after vaccination with GM-CSF-transduced allogeneic prostate cancer cells. OncoImmunology.

[98] Carrell R.K., Stanton R.A., Ethier S.P., LaRue A.C., Soloff A.C. (2018). ICOSL-augmented adenoviral-based vaccination induces a bipolar Th17/Th1 T cell response against unglycosylated MUC1 antigen. Vaccine.

[99] Vonderheide R.H. (2020). CD40 Agonist Antibodies in Cancer Immunotherapy. Annu. Rev. Med..

[100] Lau S.P., Van Montfoort N., Kinderman P., Lukkes M., Klaase L., Van Nimwegen M., Van Gulijk M., Dumas J., Mustafa D.A.M., A Lievense S.L. (2020). Dendritic cell vaccination and CD40-agonist combination therapy licenses T cell-dependent antitumor immunity in a pancreatic carcinoma murine model. J. Immunother. Cancer.

[101] Van Audenaerde J.R., Marcq E., Von Scheidt B., Davey A.S., Oliver A.J., De Waele J., Quatannens D., Van Loenhout J., Pauwels P., Roeyen G. (2020). Novel combination immunotherapy for pancreatic cancer: Potent anti-tumor effects with CD40 agonist and interleukin-15 treatment. Clin. Transl. Immunol..

[102] Starzer A.M., Berghoff A.S. (2019). New emerging targets in cancer immunotherapy: CD27 (TNFRSF7). ESMO Open.

[103] Burris H.A., Infante J.R., Ansell S.M., Nemunaitis J.J., Weiss G.R., Villalobos V.M., Sikic B.I., Taylor M.H., Northfelt D.W., Carson I.W.E. (2017). Safety and Activity of Varlilumab, a Novel and First-in-Class Agonist Anti-CD27 Antibody, in Patients With Advanced Solid Tumors. J. Clin. Oncol..

[104] Ryan M.C., Kostner H., A Gordon K., Duniho S., Sutherland M.K., Yu C., Kim K.M., Nesterova A., Anderson M., A McEarchern J. (2010). Targeting pancreatic and ovarian carcinomas using the auristatin-based anti-CD70 antibody–drug conjugate SGN-75. Br. J. Cancer.

[105] Claus C., Riether C., Schürch C., Matter M., Hilmenyuk T., Ochsenbein A. (2012). CD27 Signaling Increases the Frequency of Regulatory T Cells and Promotes Tumor Growth. Cancer Res..

[106] Aftimos P., Rolfo C.C., Rottey S.S., Offner F., Bron D., Maerevoet M., Soria J.-C., Moshir M.M., Dreier T.T., Van Rompaey L.L. (2017). Phase I Dose-Escalation Study of the Anti-CD70 Antibody ARGX-110 in Advanced Malignancies. Clin. Cancer Res..

[107] Jacobs J., Deschoolmeester V., Rolfo C., Zwaenepoel K., Bossche J.V.D., Deben C., Silence K., De Haard H., Hermans C., Rottey S. (2017). Preclinical data on the combination of cisplatin and anti-CD70 therapy in non-small cell lung cancer as an excellent match in the era of combination therapy. Oncotarget.

[108] Eric L., Yeo C.J., Lillemoe K.D., Biedrzycki B., Kobrin B., Herman J., Sugar E., Piantadosi S., Cameron J.L., Solt S. (2011). A Lethally Irradiated Allogeneic Granulocyte-Macrophage Colony Stimulating Factor-Secreting Tumor Vaccine for Pancreatic Adenocarcinoma. Ann. Surg..

[109] Le D.T., Wang-Gillam A., Picozzi V., Greten T.F., Crocenzi T., Springett G., Morse M., Zeh H., Cohen D., Fine R.L. (2015). Safety and Survival With GVAX Pancreas Prime and Listeria Monocytogenes–Expressing Mesothelin (CRS-207) Boost Vaccines for Metastatic Pancreatic Cancer. J. Clin. Oncol..

[110] Le D.T., Picozzi V.J., Ko A.H., Wainberg Z.A., Kindler H., Wang-Gillam A., Oberstein P.E., Morse M.A., Zeh H.J., Weekes C.D. (2019). Results from a Phase IIb, Randomized, Multicenter Study of GVAX Pancreas and CRS-207 Compared with Chemotherapy in Adults with Previously Treated Metastatic Pancreatic Adenocarcinoma (ECLIPSE Study). Clin. Cancer Res..

[111] Tsujikawa T., Crocenzi T., Durham J.N., Sugar E.A., Wu A.A., Onners B., Nauroth J.M., Anders R.A., Fertig E.J., Laheru D.A. (2020). Evaluation of Cyclophosphamide/GVAX Pancreas Followed by Listeria-Mesothelin (CRS-207) with or without Nivolumab in Patients with Pancreatic Cancer. Clin. Cancer Res..

[112] Wu A.A., Bever K.M., Ho W.J., Fertig E.J., Niu N., Zheng L., Parkinson R.M., Durham J.N., Onners B.L., Ferguson A.K. (2020). A Phase II Study of Allogeneic GM-CSF–Transfected Pancreatic Tumor Vaccine (GVAX) with Ipilimumab as Maintenance Treatment for Metastatic Pancreatic Cancer. Clin. Cancer Res..

[113] Wedén S., Klemp M., Gladhaug I.P., Møller M., Eriksen J.A., Gaudernack G., Buanes T. (2010). Long-term follow-up of patients with resected pancreatic cancer following vaccination against mutant K-ras. Int. J. Cancer.

[114] Palmer D.H., Valle J.W., Ma Y.T., Faluyi O., Neoptolemos J.P., Gjertsen T.J., Iversen B., Eriksen J.A., Møller A.-S., Aksnes A.-K. (2020). TG01/GM-CSF and adjuvant gemcitabine in patients with resected RAS-mutant adenocarcinoma of the pancreas (CT TG01-01): A single-arm, phase 1/2 trial. Br. J. Cancer.

[115] Middleton G., Silcocks P., Cox T., Valle J., Wadsley J., Propper D., Coxon F., Ross P., Madhusudan S., Roques T. (2014). Gemcitabine and capecitabine with or without telomerase peptide vaccine GV1001 in patients with locally advanced or metastatic pancreatic cancer (TeloVac): An open-label, randomised, phase 3 trial. Lancet Oncol..

[116] Caprotti R., Brivio F., Fumagalli L., Nobili C., Degrate L., Lissoni P., Parolini D., Messina G., Colciago M., Scotti M. (2008). Free-from-progression period and overall short preoperative immunotherapy with IL-2 increases the survival of pancreatic cancer patients treated with macroscopically radical surgery. Anticancer. Res..

[117] Lygidakis N.J., E Berberabe A., Spentzouris N., Dedemadi G., Kalligas T., Loukas G., Sotiropoulou V. (1999). A prospective randomized study using adjuvant locoregional chemoimmunotherapy in combination with surgery for pancreatic carcinoma. Hepatogastroenterology.

[118] Circelli L., Tornesello M.L., Buonaguro F.M., Buonaguro L. (2017). Use of adjuvants for immunotherapy. Hum. Vaccines Immunother..

[119] Khong H., Overwijk W.W. (2016). Adjuvants for peptide-based cancer vaccines. J. Immunother. Cancer.

[120] Fransen M.F., van der Sluis T., Ossendorp F., Arens R., Melief C.J. (2013). Controlled Local Delivery of CTLA-4 Blocking Antibody Induces CD8+ T-Cell–Dependent Tumor Eradication and Decreases Risk of Toxic Side Effects. Clin. Cancer Res..

[121] Francis D.M., Manspeaker M.P., Schudel A., Sestito L.F., O’Melia M.J., Kissick H.T., Pollack B.P., Waller E.K., Thomas S.N. (2020). Blockade of immune checkpoints in lymph nodes through locoregional delivery augments cancer immunotherapy. Sci. Transl. Med..

[122] Nierkens S., Brok M.H.D., Roelofsen T., Wagenaars J.A.L., Figdor C.G., Ruers T.J., Adema G.J. (2009). Route of Administration of the TLR9 Agonist CpG Critically Determines the Efficacy of Cancer Immunotherapy in Mice. PLoS ONE.

[123] Krieg A.M. (2008). Toll-like receptor 9 (TLR9) agonists in the treatment of cancer. Oncogene.

[124] Smits E., Ponsaerts P., Berneman Z., Van Tendeloo V. (2008). The Use of TLR7 and TLR8 Ligands for the Enhancement of Cancer Immunotherapy. Oncology.

[125] Dajon M., Iribarren K., Cremer I. (2017). Toll-like receptor stimulation in cancer: A pro- and anti-tumor double-edged sword. Immunobiol..

[126] Leppänen J., Helminen O., Huhta H., Kauppila J.H., Isohookana J., Haapasaari K.-M., Lehenkari P., Saarnio J., Karttunen T.J. (2017). High toll-like receptor (TLR) 9 expression is associated with better prognosis in surgically treated pancreatic cancer patients. Virchows Arch..

[127] Lanki M., Seppänen H., Mustonen H., Hagström J., Haglund C. (2019). Toll-like receptor 1 predicts favorable prognosis in pancreatic cancer. PLoS ONE.

[128] A Lanki M., E Seppänen H., Mustonen H.K., Böckelman C., Juuti A.T., Hagström J., Haglund C.H. (2018). Toll-like receptor 2 and Toll-like receptor 4 predict favorable prognosis in local pancreatic cancer. Tumor Biol..

[129] Zambirinis C., Levie E., Nguy S., Avanzi A., Barilla R., Xu Y., Seifert L., Daley D., Greco S.H., Deutsch M. (2015). TLR9 ligation in pancreatic stellate cells promotes tumorigenesis. J. Exp. Med..

[130] Sun Y., Wu C., Ma J., Yang Y., Man X., Wu H., Li S. (2016). Toll-like receptor 4 promotes angiogenesis in pancreatic cancer via PI3K/AKT signaling. Exp. Cell Res..

[131] Pandey S., Singh S., Anang V., Bhatt A.N., Natarajan K., Dwarakanath B.S. (2015). Pattern Recognition Receptors in Cancer Progression and Metastasis. Cancer Growth Metastasis.

[132] Jacobs C., Duewell P., Heckelsmiller K., Wei J., Bauernfeind F., Ellermeier J., Kisser U., Bauer C.A., Dauer M., Eigler A. (2010). An ISCOM vaccine combined with a TLR9 agonist breaks immune evasion mediated by regulatory T cells in an orthotopic model of pancreatic carcinoma. Int. J. Cancer.

[133] Michaelis K.A., Norgard M.A., Zhu X., Levasseur P.R., Sivagnanam S., Liudahl S.M., Burfeind K.G., Olson B., Pelz K.R., Ramos D.M.A. (2019). The TLR7/8 agonist R848 remodels tumor and host responses to promote survival in pancreatic cancer. Nat. Commun..

[134] Zou B.-B., Wang F., Li L., Cheng F.-W., Jin R., Luo X., Zhu L.-X., Geng X., Zhang S.-Q. (2015). Activation of Toll-like receptor 7 inhibits the proliferation and migration, and induces the apoptosis of pancreatic cancer cells. Mol. Med. Rep..

[135] Pratesi G., Petrangolini G., Tortoreto M., Addis A., Belluco S., Rossini A., Selleri S., Rumio C., Menard S., Balsari A. (2005). Therapeutic Synergism of Gemcitabine and CpG-Oligodeoxynucleotides in an Orthotopic Human Pancreatic Carcinoma Xenograft. Cancer Res..

[136] Schölch S., Rauber C., Tietz A., Rahbari N.N., Bork U., Schmidt T., Kahlert C., Haberkorn U., Tomai M.A., Lipson K. (2014). Radiotherapy combined with TLR7/8 activation induces strong immune responses against gastrointestinal tumors. Oncotarget.

[137] Narayanan J.S.S., Ray P., Hayashi T., Whisenant T.C., Vicente D., Carson D.A., Miller A.M., Schoenberger S.P., White R.R. (2019). Irreversible Electroporation Combined with Checkpoint Blockade and TLR7 Stimulation Induces Antitumor Immunity in a Murine Pancreatic Cancer Model. Cancer Immunol. Res..

[138] Lorkowski M., Atukorale P., Bielecki P., Tong K., Covarrubias G., Zhang Y., Loutrianakis G., Moon T., Santulli A., Becicka W. (2020). Immunostimulatory nanoparticle incorporating two immune agonists for the treatment of pancreatic tumors. J. Control. Release.

[139] Geboers B., Timmer F., Ruarus A., Pouw J., Schouten E., Bakker J., Puijk R., Nieuwenhuizen S., Dijkstra M., Tol M.V.D. (2021). Irreversible Electroporation and Nivolumab Combined with Intratumoral Administration of a Toll-Like Receptor Ligand, as a Means of In Vivo Vaccination for Metastatic Pancreatic Ductal Adenocarcinoma (PANFIRE-III). A Phase-I Study Protocol. Cancers.

[140] Jiang M., Chen P., Wang L., Li W., Chen B., Liu Y., Wang H., Zhao S., Ye L., He Y. (2020). cGAS-STING, an important pathway in cancer immunotherapy. J. Hematol. Oncol..

[141] Jing W., McAllister D., Vonderhaar E.P., Palen K., Riese M.J., Gershan J., Johnson B.D., Dwinell M.B. (2019). STING agonist inflames the pancreatic cancer immune microenvironment and reduces tumor burden in mouse models. J. Immunother. Cancer.

[142] Kinkead H.L., Hopkins A., Lutz E., Wu A.A., Yarchoan M., Cruz K., Woolman S., Vithayathil T., Glickman L.H., Ndubaku C.O. (2018). Combining STING-based neoantigen-targeted vaccine with checkpoint modulators enhances antitumor immunity in murine pancreatic cancer. JCI Insight.

[143] Foote J.B., Kok M., Leatherman J.M., Armstrong T.D., Marcinkowski B., Ojalvo L.S., Kanne D.B., Jaffee E., Dubensky T.W., Emens L.A. (2017). A STING Agonist Given with OX40 Receptor and PD-L1 Modulators Primes Immunity and Reduces Tumor Growth in Tolerized Mice. Cancer Immunol. Res..

[144] Baird J.R., Friedman D., Cottam B., Dubensky T.W., Kanne D.B., Bambina S., Bahjat K.S., Crittenden M.R., Gough M.J. (2015). Radiotherapy Combined with Novel STING-Targeting Oligonucleotides Results in Regression of Established Tumors. Cancer Res..

[145] Gogoi H., Mansouri S., Jin L. (2020). The Age of Cyclic Dinucleotide Vaccine Adjuvants. Vaccines.

[146] Geddes K., Magalhães J.G., Girardin S.E. (2009). Unleashing the therapeutic potential of NOD-like receptors. Nat. Rev. Drug Discov..

[147] Hemminki O., Dos Santos J.M., Hemminki A. (2020). Oncolytic viruses for cancer immunotherapy. J. Hematol. Oncol..

[148] de Graaf J., de Vor L., Fouchier R., Hoogen B.V.D. (2018). Armed oncolytic viruses: A kick-start for antitumor immunity. Cytokine Growth Factor Rev..

[149] Zhang L., Wang W., Wang R., Zhang N., Shang H., Bi Y., Chen D., Zhang C., Li L., Yin J. (2020). Reshaping the Immune Microenvironment by Oncolytic Herpes Simplex Virus in Murine Pancreatic Ductal Adenocarcinoma. Mol. Ther..

[150] Watanabe K., Luo Y., Da T., Guedan S., Ruella M., Scholler J., Keith B., Young R.M., Engels B., Sorsa S. (2018). Pancreatic cancer therapy with combined mesothelin-redirected chimeric antigen receptor T cells and cytokine-armed oncolytic adenoviruses. JCI Insight.

[151] Nakao A., Kasuya H., Sahin T.T., Nomura N., Kanzaki A., Misawa M., Shirota T., Yamada S., Fujii T., Sugimoto H. (2010). A phase I dose-escalation clinical trial of intraoperative direct intratumoral injection of HF10 oncolytic virus in non-resectable patients with advanced pancreatic cancer. Cancer Gene Ther..

[152] Kasuya H., Kodera Y., Nakao A., Yamamura K., Gewen T., Zhiwen W., Hotta Y., Yamada S., Fujii T., Fukuda S. (2014). Phase I Dose-escalation Clinical Trial of HF10 Oncolytic Herpes Virus in 17 Japanese Patients with Advanced Cancer. Hepatogastroenterology.

[153] Aguilar L.K., Shirley L., Chung V.M., Marsh C., Walker J., Coyle W., Marx H., Bekaii-Saab T., Lesinski G.B., Swanson B. (2015). Gene-mediated cytotoxic immunotherapy as adjuvant to surgery or chemoradiation for pancreatic adenocarcinoma. Cancer Immunol. Immunother..

[154] Noonan A., Farren M., Geyer S.M., Huang Y., Tahiri S., Ahn D., Mikhail S., Ciombor K.K., Pant S., Aparo S. (2016). Randomized Phase 2 Trial of the Oncolytic Virus Pelareorep (Reolysin) in Upfront Treatment of Metastatic Pancreatic Adenocarcinoma. Mol. Ther..

[155] Hirooka Y., Kasuya H., Ishikawa T., Kawashima H., Ohno E., Villalobos I.B., Naoe Y., Ichinose T., Koyama N., Tanaka M. (2018). A Phase I clinical trial of EUS-guided intratumoral injection of the oncolytic virus, HF10 for unresectable locally advanced pancreatic cancer. BMC Cancer.

[156] Hajda J., Lehmann M., Krebs O., Kieser M., Geletneky K., Jäger D., Dahm M., Huber B., Schöning T., Sedlaczek O. (2017). A non-controlled, single arm, open label, phase II study of intravenous and intratumoral administration of ParvOryx in patients with metastatic, inoperable pancreatic cancer: ParvOryx02 protocol. BMC Cancer.

[157] Beatty G.L., O’Hara M., Lacey S.F., Torigian D.A., Nazimuddin F., Chen F., Kulikovskaya I.M., Soulen M.C., McGarvey M., Nelson A.M. (2018). Activity of Mesothelin-Specific Chimeric Antigen Receptor T Cells Against Pancreatic Carcinoma Metastases in a Phase 1 Trial. Gastroenterology.

[158] Aoki T., Matsushita H., Hoshikawa M., Hasegawa K., Kokudo N., Kakimi K. (2017). Adjuvant combination therapy with gemcitabine and autologous γδ T-cell transfer in patients with curatively resected pancreatic cancer. Cytotherapy.

[159] Kumai T., Mizukoshi E., Hashiba T., Nakagawa H., Kitahara M., Miyashita T., Mochizuki T., Goto S., Kamigaki T., Takimoto R. (2020). Effect of adoptive T-cell immunotherapy on immunological parameters and prognosis in patients with advanced pancreatic cancer. Cytotherapy.

[160] Valilou S.F., Rezaei N., Rezaei N., Keshavarz-Fathi M. (2019). Chapter 4—Tumor Antigens. Vaccines for Cancer Immunotherapy.

[161] Haen S.P., Löffler M.W., Rammensee H.-G., Brossart P. (2020). Towards new horizons: Characterization, classification and implications of the tumour antigenic repertoire. Nat. Rev. Clin. Oncol..

[162] Zhang R., Billingsley M., Mitchell M.J. (2018). Biomaterials for vaccine-based cancer immunotherapy. J. Control. Release.

[163] Kaida M., Morita-Hoshi Y., Soeda A., Wakeda T., Yamaki Y., Kojima Y., Ueno H., Kondo S., Morizane C., Ikeda M. (2011). Phase 1 Trial of Wilms Tumor 1 (WT1) Peptide Vaccine and Gemcitabine Combination Therapy in Patients With Advanced Pancreatic or Biliary Tract Cancer. J. Immunother..

[164] Nishida S., Koido S., Takeda Y., Homma S., Komita H., Takahara A., Morita S., Ito T., Morimoto S., Hara K. (2014). Wilms Tumor Gene (WT1) Peptide–based Cancer Vaccine Combined With Gemcitabine for Patients With Advanced Pancreatic Cancer. J. Immunother..

[165] Koido S., Homma S., Okamoto M., Takakura K., Mori M., Yoshizaki S., Tsukinaga S., Odahara S., Koyama S., Imazu H. (2014). Treatment with Chemotherapy and Dendritic Cells Pulsed with Multiple Wilms’ Tumor 1 (WT1)–Specific MHC Class I/II–Restricted Epitopes for Pancreatic Cancer. Clin. Cancer Res..

[166] Tsukinaga S., Kajihara M., Takakura K., Ito Z., Kanai T., Saito K., Takami S., Kobayashi H., Matsumoto Y., Odahara S. (2015). Prognostic significance of plasma interleukin-6/-8 in pancreatic cancer patients receiving chemoimmunotherapy. World J. Gastroenterol..

[167] Mayanagi S., Kitago M., Sakurai T., Matsuda T., Fujita T., Higuchi H., Taguchi J., Takeuchi H., Itano O., Aiura K. (2015). Phase I pilot study of Wilms tumor gene 1 peptide-pulsed dendritic cell vaccination combined with gemcitabine in pancreatic cancer. Cancer Sci..

[168] Yanagisawa R., Koizumi T., Koya T., Sano K., Koido S., Nagai K., Kobayashi M., Okamoto M., Sugiyama H., Shimodaira S. (2018). WT1-pulsed Dendritic Cell Vaccine Combined with Chemotherapy for Resected Pancreatic Cancer in a Phase I Study. Anticancer. Res..

[169] Nishida S., Ishikawa T., Egawa S., Koido S., Yanagimoto H., Ishii J., Kanno Y., Kokura S., Yasuda H., Oba M.S. (2018). Combination Gemcitabine and WT1 Peptide Vaccination Improves Progression-Free Survival in Advanced Pancreatic Ductal Adenocarcinoma: A Phase II Randomized Study. Cancer Immunol. Res..

[170] Hanada S., Tsuruta T., Haraguchi K., Okamoto M., Sugiyama H., Koido S. (2018). Long-term survival of pancreatic cancer patients treated with multimodal therapy combined with WT1-targeted dendritic cell vaccines. Hum. Vaccines Immunother..

[171] Nagai K., Adachi T., Harada H., Eguchi S., Sugiyama H., Miyazaki Y. (2020). Dendritic Cell-based Immunotherapy Pulsed With Wilms Tumor 1 Peptide and Mucin 1 as an Adjuvant Therapy for Pancreatic Ductal Adenocarcinoma After Curative Resection: A Phase I/IIa Clinical Trial. Anticancer. Res..

[172] Asahara S., Takeda K., Yamao K., Maguchi H., Yamaue H. (2013). Phase I/II clinical trial using HLA-A24-restricted peptide vaccine derived from KIF20A for patients with advanced pancreatic cancer. J. Transl. Med..

[173] Suzuki N., Hazama S., Ueno T., Matsui H., Shindo Y., Iida M., Yoshimura K., Yoshino S., Takeda K., Oka M. (2014). A Phase I Clinical Trial of Vaccination With KIF20A-derived Peptide in Combination with Gemcitabine for Patients with Advanced Pancreatic Cancer. J. Immunother..

[174] Miyazawa M., Ohsawa R., Tsunoda T., Hirono S., Kawai M., Tani M., Nakamura Y., Yamaue H. (2010). Phase I clinical trial using peptide vaccine for human vascular endothelial growth factor receptor 2 in combination with gemcitabine for patients with advanced pancreatic cancer. Cancer Sci..

[175] Yamaue H., Tsunoda T., Tani M., Miyazawa M., Yamao K., Mizuno N., Okusaka T., Ueno H., Boku N., Fukutomi A. (2015). Randomized phase II/III clinical trial of elpamotide for patients with advanced pancreatic cancer: PEGASUS—PC Study. Cancer Sci..

[176] Suzuki N., Hazama S., Iguchi H., Uesugi K., Tanaka H., Hirakawa K., Aruga A., Hatori T., Ishizaki H., Umeda Y. (2016). Phase II clinical trial of peptide cocktail therapy for patients with advanced pancreatic cancer: VENUS-PC study. Cancer Sci..

[177] Miyazawa M., Katsuda M., Maguchi H., Katanuma A., Ishii H., Ozaka M., Yamao K., Imaoka H., Kawai M., Hirono S. (2016). Phase II clinical trial using novel peptide cocktail vaccine as a postoperative adjuvant treatment for surgically resected pancreatic cancer patients. Int. J. Cancer.

[178] Kameshima H., Tsuruma T., Kutomi G., Shima H., Iwayama Y., Kimura Y., Imamura M., Torigoe T., Takahashi A., Hirohashi Y. (2012). Immunotherapeutic benefit of α-interferon (IFNα) in survivin2B-derived peptide vaccination for advanced pancreatic cancer patients. Cancer Sci..

[179] Shima H., Tsurita G., Wada S., Hirohashi Y., Yasui H., Hayashi H., Miyakoshi T., Watanabe K., Murai A., Asanuma H. (2019). Randomized phase II trial of survivin 2B peptide vaccination for patients with HLA -A24-positive pancreatic adenocarcinoma. Cancer Sci..

[180] Rong Y., Qin X., Jin D., Lou W., Wu L., Wang D., Wu W., Ni X., Mao Z., Kuang T. (2011). A phase I pilot trial of MUC1-peptide-pulsed dendritic cells in the treatment of advanced pancreatic cancer. Clin. Exp Med..

[181] Le D.T., Brockstedt D.G., Nir-Paz R., Hampl J., Mathur S., Nemunaitis J., Sterman D., Hassan R., Lutz E., Moyer B. (2011). A Live-Attenuated Listeria Vaccine (ANZ-100) and a Live-Attenuated Listeria Vaccine Expressing Mesothelin (CRS-207) for Advanced Cancers: Phase I Studies of Safety and Immune Induction. Clin. Cancer Res..

[182] Abou-Alfa G.K., Chapman P.B., Feilchenfeldt J., Brennan M., Capanu M., Gansukh B., Jacobs G., Levin A., Neville D., Kelsen D.P. (2011). Targeting Mutated K-ras in Pancreatic Adenocarcinoma Using an Adjuvant Vaccine. Am. J. Clin. Oncol..

[183] Kubuschok B., Pfreundschuh M., Breit R., Hartmann F., Sester M., Gärtner B., König J., Murawski N., Held G., Zwick C. (2012). Mutated Ras-Transfected, EBV-Transformed Lymphoblastoid Cell Lines as a Model Tumor Vaccine for Boosting T-Cell Responses Against Pancreatic Cancer: A Pilot Trial. Hum. Gene Ther..

[184] Bassani-Sternberg M., Digklia A., Huber F., Wagner D., Sempoux C., Stevenson B.J., Thierry A.-C., Michaux J., Pak H., Racle J. (2019). A Phase Ib Study of the Combination of Personalized Autologous Dendritic Cell Vaccine, Aspirin, and Standard of Care Adjuvant Chemotherapy Followed by Nivolumab for Resected Pancreatic Adenocarcinoma—A Proof of Antigen Discovery Feasibility in Three Patients. Front. Immunol..

[185] Noguchi M., Yanagimoto H., Shiomi H., Satoi S., Mine T., Toyokawa H., Yamamoto T., Tani T., Yamada A., Kwon A.-H. (2010). A phase II study of personalized peptide vaccination combined with gemcitabine for non-resectable pancreatic cancer patients. Oncol. Rep..

[186] Bauer C., Dauer M., Saraj S., Schnurr M., Bauernfeind F., Sterzik A., Junkmann J., Jakl V., Kiefl R., Oduncu F. (2011). Dendritic cell-based vaccination of patients with advanced pancreatic carcinoma: Results of a pilot study. Cancer Immunol. Immunother..

[187] Kimura Y., Tsukada J., Tomoda T., Takahashi H., Imai K., Shimamura K., Sunamura M., Yonemitsu Y., Shimodaira S., Koido S. (2012). Clinical and Immunologic Evaluation of Dendritic Cell–Based Immunotherapy in Combination With Gemcitabine and/or S-1 in Patients With Advanced Pancreatic Carcinoma. Pancreas.

[188] Yutani S., Komatsu N., Yoshitomi M., Matsueda S., Yonemoto K., Mine T., Noguchi M., Ishihara Y., Yamada A., Itoh K. (2013). A phase II study of a personalized peptide vaccination for chemotherapy-resistant advanced pancreatic cancer patients. Oncol. Rep..

[189] Qiu Y., Yun M.M., Xu M.B., Wang Y.Z., Yun S. (2012). Pancreatic carcinoma-specific immunotherapy using synthesised alpha-galactosyl epitope-activated immune responders: Findings from a pilot study. Int. J. Clin. Oncol..

[190] Lin M., Yuan Y.-Y., Liu S.-P., Shi J.-J., Long X.-A., Niu L.-Z., Chen J.-B., Li Q., Xu K.-C. (2015). Prospective study of the safety and efficacy of a pancreatic cancer stem cell vaccine. J. Cancer Res. Clin. Oncol..

[191] Zhang L., Zhu W., Li J., Yang X., Ren Y., Niu J., Pang Y. (2015). Clinical outcome of immunotherapy with dendritic cell vaccine and cytokine-induced killer cell therapy in hepatobiliary and pancreatic cancer. Mol. Clin. Oncol..

[192] Mehrotra S., Britten C.D., Chin S., Garrett-Mayer E., Cloud C.A., Li M., Scurti G., Salem M., Nelson M.H., Thomas M.B. (2017). Vaccination with poly(IC:LC) and peptide-pulsed autologous dendritic cells in patients with pancreatic cancer. J. Hematol. Oncol..

[193] Ushach I., Zlotnik A. (2016). Biological role of granulocyte macrophage colony-stimulating factor (GM-CSF) and macrophage colony-stimulating factor (M-CSF) on cells of the myeloid lineage. J. Leukoc. Biol..

[194] Cheever M.A., Allison J., Ferris A.S., Finn O.J., Hastings B.M., Hecht T.T., Mellman I., Prindiville S.A., Viner J.L., Weiner L.M. (2009). The Prioritization of Cancer Antigens: A National Cancer Institute Pilot Project for the Acceleration of Translational Research. Clin. Cancer Res..

[195] Oji Y., Nakamori S., Fujikawa M., Nakatsuka S.-I., Yokota A., Tatsumi N., Abeno S., Ikeba A., Takashima S., Tsujie M. (2004). Overexpression of the Wilms’ tumor gene WT1 in pancreatic ductal adenocarcinoma. Cancer Sci..

[196] Koido S., Okamoto M., Kobayashi M., Shimodaira S., Sugiyama H. (2017). Significance of Wilms’ tumor 1 antigen as a cancer vaccine for pancreatic cancer. Discov. Med..

[197] Taniuchi K., Nakagawa H., Nakamura T., Eguchi H., Ohigashi H., Ishikawa O., Katagiri T., Nakamura Y. (2005). Down-regulation of RAB6KIFL/KIF20A, a kinesin involved with membrane trafficking of discs large homologue 5, can attenuate growth of pancreatic cancer cell. Cancer Res..

[198] Imai K., Hirata S., Irie A., Senju S., Ikuta Y., Yokomine K., Harao M., Inoue M., Tomita Y., Tsunoda T. (2010). Identification of HLA-A2-restricted CTL epitopes of a novel tumour-associated antigen, KIF20A, overexpressed in pancreatic cancer. Br. J. Cancer.

[199] Rapisarda A., Melillo G. (2012). Role of the VEGF/VEGFR Axis in Cancer Biology and Therapy. Adv. Cancer Res..

[200] Seo Y., Baba H., Fukuda T., Takashima M., Sugimachi K. (2000). High expression of vascular endothelial growth factor is associated with liver metastasis and a poor prognosis for patients with ductal pancreatic adenocarcinoma. Cancer.

[201] Liang Q.-L., Wang B.-R., Chen G.-Q., Li G.-H., Xu Y.-Y. (2009). Clinical significance of vascular endothelial growth factor and connexin43 for predicting pancreatic cancer clinicopathologic parameters. Med. Oncol..

[202] Dong H., Qian N., Wang Y., Meng L., Chen D., Ji X., Feng W. (2015). Survivin expression and serum levels in pancreatic cancer. World J. Surg. Oncol..

[203] Brown M., Zhang W., Yan D., Kenath R., Le L.T.T., Wang H., Delitto D., Ostrov D., Robertson K., Liu C. (2020). The role of survivin in the progression of pancreatic ductal adenocarcinoma (PDAC) and a novel survivin-targeted therapeutic for PDAC. PLoS ONE.

[204] Suh H., Pillai K., Morris D.L. (2017). Mucins in pancreatic cancer: Biological role, implications in carcinogenesis and applications in diagnosis and therapy. Am. J. Cancer Res..

[205] Lv J., Li P. (2019). Mesothelin as a biomarker for targeted therapy. Biomark. Res..

[206] Chen S.-H., Hung W.-C., Wang P., Paul C., Konstantopoulos K. (2013). Mesothelin Binding to CA125/MUC16 Promotes Pancreatic Cancer Cell Motility and Invasion via MMP-7 Activation. Sci. Rep..

[207] Jafri M.A., Ansari S.A., Alqahtani M.H., Shay J.W. (2016). Roles of telomeres and telomerase in cancer, and advances in telomerase-targeted therapies. Genome Med..

[208] Kim J., Reber H.A., Dry S.M., Elashoff D., Chen S.L., Umetani N., Kitago M., Hines O.J., Kazanjian K.K., Hiramatsu S. (2006). Unfavourable prognosis associated with K-ras gene mutation in pancreatic cancer surgical margins. Gut.

[209] Uesaka K., Boku N., Fukutomi A., Okamura Y., Konishi M., Matsumoto I., Kaneoka Y., Shimizu Y., Nakamori S., Sakamoto H. (2016). Adjuvant chemotherapy of S-1 versus gemcitabine for resected pancreatic cancer: A phase 3, open-label, randomised, non-inferiority trial (JASPAC 01). Lancet.

[210] Neoptolemos J.P., Stocken D.D., Bassi C., Ghaneh P., Cunningham D., Goldstein D., Padbury R., Moore M.J., Gallinger S., Mariette C. (2010). Adjuvant Chemotherapy With Fluorouracil Plus Folinic Acid vs. Gemcitabine Following Pancreatic Cancer Resection. JAMA.

[211] Oettle H., Neuhaus P., Hochhaus A., Hartmann J.T., Gellert K., Ridwelski K., Niedergethmann M., Zülke C., Fahlke J., Arning M.B. (2013). Adjuvant Chemotherapy With Gemcitabine and Long-term Outcomes Among Patients With Resected Pancreatic Cancer. JAMA.

[212] Van Laethem J.-L., Hammel P., Mornex F., Azria D., Van Tienhoven G., Vergauwe P., Peeters M., Polus M., Praet M., Mauer M. (2010). Adjuvant Gemcitabine Alone Versus Gemcitabine-Based Chemoradiotherapy After Curative Resection for Pancreatic Cancer: A Randomized EORTC-40013-22012/FFCD-9203/GERCOR Phase II Study. J. Clin. Oncol..

[213] Ott P.A., Shuqiang L., Keskin D.B., Shukla S.A., Sun J., Bozym D.J., Zhang W., Luoma A., Giobbie-Hurder A., Peter L. (2017). An immunogenic personal neoantigen vaccine for patients with melanoma. Nat. Cell Biol..

[214] Ott P.A., Hu-Lieskovan S., Chmielowski B., Govindan R., Naing A., Bhardwaj N., Margolin K., Awad M.M., Hellmann M.D., Lin J.J. (2020). A Phase Ib Trial of Personalized Neoantigen Therapy Plus Anti-PD-1 in Patients with Advanced Melanoma, Non-small Cell Lung Cancer, or Bladder Cancer. Cell.

[215] Keskin D.B., Anandappa A., Sun J., Tirosh I., Mathewson N., Li S., Oliveira G., Giobbie-Hurder A., Felt K., Gjini E. (2018). Neoantigen vaccine generates intratumoral T cell responses in phase Ib glioblastoma trial. Nat. Cell Biol..

[216] Blass E., Ott P.A. (2021). Advances in the development of personalized neoantigen-based therapeutic cancer vaccines. Nat. Rev. Clin. Oncol..

[217] Murakami T., Homma Y., Matsuyama R., Mori R., Miyake K., Tanaka Y., Den K., Nagashima Y., Nakazawa M., Hiroshima Y. (2017). Neoadjuvant chemoradiotherapy of pancreatic cancer induces a favorable immunogenic tumor microenvironment associated with increased major histocompatibility complex class I-related chain A/B expression. J. Surg. Oncol..

[218] Carvalho R.C.V.H.D.A., Villar R.C. (2018). Radiotherapy and immune response: The systemic effects of a local treatment. Clinics.

[219] Lin M., Liang S., Wang X., Liang Y., Zhang M., Chen J., Niu L., Xu K. (2017). Percutaneous irreversible electroporation combined with allogeneic natural killer cell immunotherapy for patients with unresectable (stage III/IV) pancreatic cancer: A promising treatment. J. Cancer Res. Clin. Oncol..

[220] Lin M., Zhang X., Liang S., Luo H., Alnaggar M., Liu A., Yin Z., Chen J., Niu L., Jiang Y. (2020). Irreversible electroporation plus allogenic Vγ9Vδ2 T cells enhances antitumor effect for locally advanced pancreatic cancer patients. Signal Transduct. Target. Ther..

[221] O’Neill C., Hayat T., Hamm J., Healey M., Zheng Q., Li Y., Martin R.C. (2020). A phase 1b trial of concurrent immunotherapy and irreversible electroporation in the treatment of locally advanced pancreatic adenocarcinoma. Surgery.

[222] Lin C., Verma V., Lazenby A., Ly Q.P., Berim L.D., Schwarz J.K., Madiyalakan M., Nicodemus C.F., Hollingsworth M.A., Meza J.L. (2019). Phase I/II Trial of Neoadjuvant Oregovomab-based Chemoimmunotherapy Followed by Stereotactic Body Radiotherapy and Nelfinavir For Locally Advanced Pancreatic Adenocarcinoma. Am. J. Clin. Oncol..

[223] Xie C., Duffy A.G., Brar G., Fioravanti S., Mabry-Hrones D., Walker M., Bonilla C.M., Wood B.J., Citrin D.E., Gil Ramirez E.M. (2020). Immune Checkpoint Blockade in Combination with Stereotactic Body Radiotherapy in Patients with Metastatic Pancreatic Ductal Adenocarcinoma. Clin. Cancer Res..

[224] Scheffer H.J., Stam A.G., Geboers B., Vroomen L.G., Ruarus A., De Bruijn B., Tol M.P.V.D., Kazemier G., Meijerink M.R., De Gruijl T.D. (2019). Irreversible electroporation of locally advanced pancreatic cancer transiently alleviates immune suppression and creates a window for antitumor T cell activation. OncoImmunology.

[225] Pandit H., Hong Y.K., Li Y., Rostas J., Pulliam Z., Li S.P., Martin R.C.G. (2019). Evaluating the Regulatory Immunomodulation Effect of Irreversible Electroporation (IRE) in Pancreatic Adenocarcinoma. Ann. Surg. Oncol..

[226] Geboers B., Scheffer H.J., Graybill P.M., Ruarus A.H., Nieuwenhuizen S., Puijk R.S., Tol P.M.V.D., Davalos R.V., Rubinsky B., De Gruijl T.D. (2020). High-Voltage Electrical Pulses in Oncology: Irreversible Electroporation, Electrochemotherapy, Gene Electrotransfer, Electrofusion, and Electroimmunotherapy. Radiology.

[227] Yang J., Eresen A., Shangguan J., Ma Q., Yaghmai V., Zhang Z. (2021). Irreversible electroporation ablation overcomes tumor-associated immunosuppression to improve the efficacy of DC vaccination in a mice model of pancreatic cancer. OncoImmunology.

[228] Zhao J., Wen X., Tian L., Li T., Xu C., Wen X., Melancon M.P., Gupta S., Shen B., Peng W. (2019). Irreversible electroporation reverses resistance to immune checkpoint blockade in pancreatic cancer. Nat. Commun..

[229] Sun S., Liu Y., He C., Hu W., Liu W., Huang X., Wu J., Xie F., Chen C., Wang J. (2021). Combining NanoKnife with M1 oncolytic virus enhances anticancer activity in pancreatic cancer. Cancer Lett..

[230] Mills B.N., Connolly K.A., Ye J., Murphy J., Uccello T., Han B.J., Zhao T., Drage M.G., Murthy A., Qiu H. (2019). Stereotactic Body Radiation and Interleukin-12 Combination Therapy Eradicates Pancreatic Tumors by Repolarizing the Immune Microenvironment. Cell Rep..

[231] Yasmin-Karim S., Bruck P.T., Moreau M., Kunjachan S., Chen G.Z., Kumar R., Grabow S., Dougan S.K., Ngwa W. (2018). Radiation and Local Anti-CD40 Generate an Effective in situ Vaccine in Preclinical Models of Pancreatic Cancer. Front. Immunol..

